# SARM suppresses TRIF, TRAF3, and IRF3/7 mediated antiviral signaling in large yellow croaker *Larimichthys crocea*

**DOI:** 10.3389/fimmu.2022.1021443

**Published:** 2023-01-13

**Authors:** Jia Xi Zhang, Ying Li, Jun Chun Tang, Kai Qing Li, Juan Juan Shen, Chang Liu, Yong Hua Jiang, Zi Ping Zhang, Yi Lei Wang, Peng Fei Zou

**Affiliations:** ^1^ Key Laboratory of Healthy Mariculture for the East China Sea, Ministry of Agriculture and Rural Affairs, Ornamental Aquarium Engineering Research Centre in University of Fujian Province, Fisheries College, Jimei University, Xiamen, Fujian, China; ^2^ Key Laboratory of Estuarine Ecological Security and Environmental Health, Tan Kah Kee College, Xiamen University, Zhangzhou, Fujian, China; ^3^ College of the Environment and Ecology, Xiamen University, Xiamen, China; ^4^ College of Marine Science, Fujian Agriculture and Forestry University, Fuzhou, Fujian, China

**Keywords:** SARM, TRIF, TRAF3, IRF3, IRF7, large yellow croaker

## Abstract

As a TIR domain-containing molecular, sterile α-and armadillo motif-containing protein (SARM) acts as an adaptor in Toll-like receptor (TLR) signaling, and also plays important roles in mediating apoptosis and neuronal injury. In the present study, the ortholog of *SARM*, named as *Lc-SARM*, was cloned and identified in large yellow croaker (*Larimichthys crocea*). The full-length ORF of *Lc-SARM* consists of 2,154 bp, encoding a protein of 717 amino acids (aa), which is comprised of an N-terminal ARM domain, two SAM domains, and a C-terminal TIR domain. Confocal microscopy revealed that *Lc*-SARM was mainly distributed in the cytoplasm, and the mRNA expression level of *Lc-SARM* was broadly distributed in all the detected organs/tissues, with the highest expression level found in the brain. The expression patterns of *Lc-SARM* could be induced in response to poly I:C, LPS, PGN stimulations, and *Pseudomonas plecoglossicida* infection. Notably, although the overexpression of *Lc*-SARM could significantly induce NF-κB, IRF3, IRF7, and type I IFN promoter activation, whereas the co-expression of *Lc-*SARM with *Lc-*TRIF, *Lc*-TRAF3, *Lc*-IRF3, or *Lc*-IRF7 significantly down-regulated the induction of NF-κB, IRF3, IRF7, or type I IFN promoter activation, and suppressed the antiviral effects as well as the downstream antiviral-related genes expression compared to the only overexpression of *Lc*-TRIF, *Lc*-TRAF3, *Lc*-IRF3, or *Lc*-IRF7. Co-immunoprecipitation (Co-IP) assays also demonstrated that *Lc-*SARM interacts separately with *Lc*-TRIF, *Lc*-TRAF3, *Lc*-IRF3, and *Lc*-IRF7. It is thus collectively suggested that *Lc*-SARM functions as a negative regulator in *Lc*-TRIF, *Lc*-TRAF3, and *Lc*-IRF3/7 involved antiviral signaling.

## 1 Introduction

Defined as the first line of host defense against invading bacteria and viruses, the innate immune system plays important roles in the host immune responses (1, 2). The initiation of the host innate immunity mainly relays on the recognitions of pathogen-associated molecular patterns (PAMPs) by the host pattern recognition receptors (PRRs), such as Toll-like receptors (TLRs), nucleotide-binding oligomerization domain (NOD)-like receptors (NLRs), retinoic acid-inducible gene (RIG)-I-like receptors (RLRs), absent in melanoma 2 (AIM2)-like receptors (ALRs), and C-type lectin-like receptors (CLRs) (3).

In mammals, TLRs are the most widely and deeply studied PRRs, which have been demonstrated to associate with seven adaptor molecules, including myeloid differentiation protein 88 (MyD88), Toll/interleukin-1 receptor (TIR) domain-containing adapter protein (TIRAP, also known as MAL), TIR-domain-containing adaptor protein inducing IFN-β (TRIF, also known as TICAM1), TRIF-related adaptor molecule (TRAM, also known as TICAM2), sterile α-and armadillo motif-containing protein (SARM, also known as SARM1), B-cell adaptor for phosphoinositide 3-kinease (BCAP), and SLP adaptor and C-terminal Src kinase (CSK) interacting membrane protein (SCIMP) (4). Except for SCIMP, those molecules associate with TLRs *via* TIR-TIR domain interactions (5), which in turn activate a range of downstream transcription factors, such as NF-κB and interferon regulatory factor 3/7 (IRF3/7), and eventually produce the interferons (IFNs) and inflammatory-related molecules to promote the host immune responses (6, 7).

SARM, the fifth discovered TIR domain-containing adaptor, contains an N-terminal armadillo repeat motif (ARM) domain, two sterile α-motif (SAM) domains and a C-terminal TIR domain (8, 9). Notably, the function of SARM was varied during the process of evolution. The ortholog of SARM in the nematode (*Caenorhabditis elegans*), named Toll-interleukin 1 (TIR-1), has been proved to function importantly in the defense against bacterial and fungi infections, even the development of the worm (10–12). However, the function of mammalian SARM has been demonstrated to act as a negative regulator in MyD88- and TRIF-dependent TLR signaling pathways through TIR-TIR interactions (13, 14), further studies have also revealed that SARM may be directly inhibited MAPK phosphorylation (15). In addition, studies in mammals showed that SARM could induce intrinsic apoptosis by the association of SARM to the mitochondria, which was dependent on the mitochondria-targeting signal sequence at the N-terminal 27 amino acids (S27) of SARM (16, 17). Recent studies in mouse indicated that SARM was necessary for inflammatory regulation and cytokine production during viral infection in the host central nervous system (18, 19), and it was also found that SARM acted as an adaptor molecule directly involved in neuronal injury (20).

Compared to the deeply understanding of the roles of SARM in mammals, functional studies of teleost SARM are still limited. Up to date, only a few studies have reported the molecular cloning and characterization of SARM in teleosts, including zebrafish (*Danio rerio*) (21, 22), grass carp (*Ctenopharyngodon idella*) (23), and mandarin fish (*Siniperca chuatsi*) (24). Notably, studies in grass carp showed that SARM could affect the expressions of the downstream molecules in TLR-dependent pathway to inhibit the IFN-I response initiated by the infection of grass carp reovirus (GCRV) (23). Another report in mandarin fish has demonstrated that teleost SARM could interact with MyD88, TRIF, and also MAL, which functionally impair the host antiviral signaling (24). These reports collectively suggested the important function of SARM in the regulation of the host immune-related signaling in teleosts.

Large yellow croaker (*Larimichthys crocea*), as a kind of important economical marine species, is widely distributed in China’s east and south coastal area. Although the large yellow croaker breeding industry has been greatly improved, the outbreaks of infectious diseases caused by bacteria (25), parasites (26), and viruses (27) are increased, which caused large economic losses. Therefore, it is important to understand the regulation of host immune responses against such invading pathogens, which is necessary for further diseases control and prevention of the fish. In this study, one ortholog of *SARM* gene, named as *Lc-SARM*, was characterized in large yellow croaker. The protein sequence as well as the genome organization of *Lc-SARM* were analyzed and compared with other vertebrates, and the phylogenetic relationship of vertebrate SARM proteins was also constructed. Furthermore, the subcellular localization, the constitutive expression patterns of *Lc-SARM* in healthy fish, and also the inductive expression profiles under various stimulations including poly I:C, LPS, PGN, and *Pseudomonas plecoglossicida* were determined. Moreover, the potential interaction of *Lc*-SARM with *Lc*-TRIF, *Lc*-TRAF3, *Lc*-IRF3, and *Lc*-IRF7, the association of such molecules in the induction of NF-κB, IRF3, IRF7, and type I IFN signaling, and the antiviral effects as well as the downstream immune-related gene expression were also analyzed, which providing new insights into the functional understanding of SARM in the regulation of the host immune responses in teleosts.

## 2 Materials and methods

### 2.1 Fish, cell culture, virus, and transfection

Healthy large yellow croakers, with weight of 60.0 ± 15.0 g and length of 18.0 ± 1.5 cm each, were purchased from Ningde Fufa Fishing Co., Ltd., Ningde, Fujian Province, China. They were cultured with commercial feed in recirculating seawater systems and kept at 24-26°C for at least two weeks before being used for the subsequent experiments (28, 29).

Human embryonic kidney 293T (HEK 293T) cells were cultivated at 37°C with Dulbecco’s Modified Eagle Medium (DMEM), which was added with 10% fetal bovine serum (FBS, Invitrogen-Gibco), 100 U/mL penicillin and streptomycin (PS) and maintained in an incubator containing 5% CO_2_ as described previously (30). Large yellow croaker muscle (LYCMS) cells were maintained in L15 Medium (Boster, USA) supplemented with 10% FBS and 100 U/mL PS at 27°C as described previously (31). *Epithelioma papulosum cyprinid* (EPC) cells were cultured at 25°C in M199 medium (Procell, Wuhan, China) containing 10% FBS and 100 U/mL PS. Spring viraemia of carp virus (SVCV), which is infective in EPC cells, was propagated in EPC cells at 25°C. The transfection of plasmids into such cells mentioned above was performed by using Lipofectamine 3000 (Invitrogen, Carlsbad, CA) following the manufacturer’s instructions.

### 2.2 Gene cloning and plasmid construction

To get the full-length open reading frame (ORF) of *SARM* ortholog in large yellow croaker, specific primers were designed according to the transcriptome data in the NCBI database (GenBank accession No. XM_010735792.3). The confirmed ORF fragment was then constructed into pcDNA3.1/*myc*-His (−) A vector (Invitrogen, Carlsbad, CA, USA) for subsequent overexpression analysis. For subcellular localization and co-immunoprecipitation assays, the amplified ORF fragment of *SARM* was also inserted into the pTurboGFP-N vector (Evrogen, Moscow, Russia) and p3xFLAG-CMV™-14 (Sigma Aldrich, St Louis, MO), respectively. Furthermore, the ORFs of the identified large yellow croaker *TRIF*, *TRAF3*, *IRF3*, and *IRF7* were also amplified and inserted into the pcDNA3.1/*myc*-His (−) A vector and pTurboGFP-N vector, respectively. All the constructed plasmids were confirmed by sequencing and Western blotting analysis. The primers along with the restriction enzyme cutting sites which used for ORFs cloning of target genes are listed in **Table 1**.

**Table 1:** Primer sequences used in this study.

Primer name	Sequence (5’-3’)	Application
*Lc*-SARM-F	ATGTTAATTTCTCTGACGCTCTTC	*Lc-*SARM ORF cloning
*Lc*-SARM-R	TTCTTTCTTTTTCTGGCCTTTGG	
pcDNA3.1-SARM-F	CCGCTCGAGCGATGGCATTAATTTCTCTGACGCTCTTC	pcDNA3.1-SARM
pcDNA3.1-SARM-R	GGGGTACCTTCTTTCTTTTTCTGGCCTTTGG	
pcDNA3.1-TRIF-F	CCGCTCGAGCGATGGCTAGCCGCGAGGGAGAAGA	pcDNA3.1-TRIF
pcDNA3.1-TRIF-R	CGGGGTACCTTGCTCATCTAAATCATCT	
pcDNA3.1-TRAF3-F	CCGCTCGAGCGATGGCTTCAGCGGGGAGGAGTGC	pcDNA3.1-TRAF3
pcDNA3.1-TRAF3-R	CGGGGTACCCGGGTCAGGAAGGTCAGA	
pcDNA3.1-IRF3-F	CCGGAATTCCAATGGCTTCTCATTCTAAACCTC	pcDNA3.1-IRF3
pcDNA3.1-IRF3-R	CCCAAGCTTGTACAGCTCCATCATCT	
pcDNA3.1-IRF7-F	CGCGGATCCATGGCTCAAAGCCCTCCCAAG	pcDNA3.1-IRF7
pcDNA3.1-IRF7-R	CGGGGTACCATAAAGCTCAGCAGCCAG	
pTurbo-SARM-F	CCGCTCGAGATGGCATTAATTTCTCTGACGCTCTTC	pTurbo-SARM-GFP
pTurbo-SARM-R	GGGGTACCGTTTCTTTCTTTTTCTGGCCTTTGG	
pTurbo-TRIF-F	CCGCTCGAGATGGCTAGCCGCGAGGGAGAAGA	pTurbo-TRIF-GFP
pTurbo-TRIF-R	CGGGGTACCGTTTGCTCATCTAAATCATCT	
pTurbo-TRAF3-F	CCGCTCGAGATGGCTTCAGCGGGGAGGAGTGC	pTurbo-TRAF3-GFP
pTurbo-TRAF3-R	CGGGGTACCGTCGGGTCAGGAAGGTCAGA	
pTurbo-IRF3-F	CCGGAATTCCAATGGCTTCTCATTCTAAACCTC	pTurbo-IRF3-GFP
pTurbo-IRF3-R	CGGGGTACCGTGTACAGCTCCATCATCT	
pTurbo-IRF7-F	CGGGGTACCGATGGCTCAAAGCCCTCCCAAG	pTurbo-IRF7-GFP
pTurbo-IRF7-R	CGCGGATCCCGATAAAGCTCAGCAGCCAG	
p3xFLAG-SARM-F	CCCAAGCTTATGGCATTAATTTCTCTGACGCTCTTC	p3xFLAG-SARM
p3xFLAG-SARM-R	GGGGTACCGTTTCTTTCTTTTTCTGGCCTTTGG	
qSARM-F	ATCAAATCTCGTCAACGAAACACC	qRT-PCR
qSARM-R	GCTCCTGGAAACGGTCACAAT	
qIFN1-F	GCTCAGCAGGATCTTGTTTGTG	qRT-PCR
qIFN1-R	CAGCTGATGCTTTGGACGC	
qIRF3-F	AAGATGGGCGATGGTTTGG	qRT-PCR
qIRF3-R	GCTCTATGGGCTGTCTGCTACTG	
qIRF7-F	ATGGGCAGTAGCAAGTGGTAAA	qRT-PCR
qIRF7-R	ACTCTGTGGGCGAGTTGTAGAT	
qMx-F	AGGATAAAATGGCGGGAAGT	qRT-PCR
qMx-R	AAAGCCTCTGTGGTTGCTATGT	
qRSAD2-F	CCCAAGTGTCAGCATCGTCA	qRT-PCR
qRSAD2-R	TGCGAATCTTGTAAAGGCAATC	
qISG56-F	GCGCGATAGAAACAGGTCAAT	qRT-PCR
qISG56-R	TGCCAGGAAGGCCTCTATTTC	
q*Lc*-β-actin-F	TTATGAAGGCTATGCCCTGCC	qRT-PCR
q*Lc*-β-actin-R	TGAAGGAGTAGCCACGCTCTGT	
qSVCV-G-F	CGACCTGGATTAGACTTG	qRT-PCR
qSVCV-G-R	AATGTTCCGTTTCTCACT	
qSVCV-M-F	TACTCCTCCCACTTACGA	qRT-PCR
qSVCV-M-R	CAAGAGTCCGAGAAGGTC	
qEPC-β-actin-F	TGTTCCAGCCATCCTTCTTG	qRT-PCR
qEPC-β-actin-R	TGATTTTCATTGTGCTGGGG	

### 2.3 Immune stimulation and qRT-PCR analysis

To analyze the mRNA expression patterns of *SARM* gene in various organs/tissues of large yellow croaker, six healthy fish were collected and anesthetized in 0.01% eugenol, and the organs/tissues including the spleen, liver, intestine, skin, brain, muscle, heart, gill, trunk kidney, and head kidney were collected and stored in RNA later, with the blood collected and added in Trizol, which were then used for subsequent total RNA extraction.

To determine the expression profiles of *SARM* under various immune stimulations, the healthy fish were equally divided into five groups (four experimental groups and a control group), with each group contained 50 fish. In the experimental groups, each fish was injected intraperitoneally with 100 µL of PBS containing polyinosinic-polycytidylic acid (poly I:C) (1 mg/mL), peptidoglycan (PGN) (1 mg/mL), lipopolysaccharides (LPS) (0.5 mg/mL), or *Pseudomonas plecoglossicida* (5 × 10^5^ CFU/mL), whereas the fish of the control group was injected intraperitoneally with 100 µL of PBS, respectively (32, 33). At 6, 12, and 24 h post-injection (hpi), six fish were randomly selected and anesthetized as described above, various organs including the head kidney, gill, and intestine were gathered for total RNA extraction.

Total RNA was isolated from the organs/tissues as well as the LYCMS and EPC cell samples by Eastep™ Super Total RNA Extraction Kit (Promega, Beijing, China) following the manufacturer’s protocol. The first-strand cDNA sequence was synthesized by using the first-stand cDNA synthesis kit (RevertAid First Stand cDNA Synthesis Kit, #K1622, Thermo Scientific™) according to the manufacturer’s instructions. The synthesized cDNA products were stored at −80°C and used as templates for target gene ORF cloning and qRT-PCR assays.

qRT-PCR assays were conducted by Roche LightCycler^®^ 480 II quantitative real-time detection system (Roche, Switzerland) with Go Taq^®^ qPCR Master Mix (Promega, Madison, WI, USA). The qRT-PCR was performed using the following program: 95°C for 5 min, followed by 40 cycles at 95°C for 20 s, 56°C for 15 s, and 72°C for 15 s. After amplification, melting curve analyses were verified by heating the sample from 65°C to 95°C, with a rate of 0.5°C per second. All reactions were conducted in a 384-well plate in triplicate. The relative expression levels of the target genes were calculated using the comparative Ct method (2^−ΔΔCt^), with the *β-actin* used as an internal reference gene (34). The primers used for qRT-PCR analysis are listed in **Table 1**.

### 2.4 Bioinformatics analysis

The predicted protein sequence was analyzed using the ExPASy Bioinformatics Resource Portal program (http://www.expasy.org/proteomics), with the conserved domains predicted by the Conserved Domain Database (CDD) on the National Center for Biotechnology Information website (NCBI, https://www.ncbi.nlm.nih.gov/cdd) (35) and the Simple Modular Architecture Research Tool (SMART) (http://smart.embl.de) (36). Orthologues of vertebrate SARM were searched by the Basic Local Alignment Search Tool (BLAST) program of NCBI (http://blast.ncbi.nlm.nih.gov/Blast.cgi). The multiple sequence alignments were created with the Clustal X software (37) and then edited by the GeneDoc program. The phylogenetic tree of vertebrate SARM was constructed using the MEGA version 7.0 with the neighbor-joining method (38). The vertebrate *SARM* gene sequences were searched on NCBI genome database (https://www.ncbi.nlm.nih.gov/genome/), and then analyzed online with the Splign (https://www.ncbi.nlm.nih.gov/sutils/splign/splign.cgi).

### 2.5 Confocal microscopy

To examine the subcellular localization of the SARM ortholog from large yellow croaker, HEK 293T cells seeded on sterilized coverslips in 6-well plates (2 × 10^5^ cells per well) were transfected with the expressing plasmid pTurbo-SARM-GFP or empty vector pTurboGFP-N (5 µg per well) by using Lipofectamine 3000 (Invitrogen). The transfected cells were washed with 1 × PBS at 24 h post-transfection (hpt), fixed with 4% paraformaldehyde, and then permeabilized with Triton X-100 (0.2% in PBS) for 10 min. The cells on the coverslips were stained with DAPI, which was performed using one drop of the mounting medium (VECTASHIELDR Hard Set™ Mounting Medium with DAPI, Vector Laboratories, CA), followed by examining and photographing under a confocal microscope (Leica TCS SP8, Germany). The cells were then harvested and lysed using the RIPA buffer (Beyotime, Shanghai, China) containing protease inhibitors (Beyotime, Shanghai, China) for the detection of *Lc*-SARM-GFP and pTurboGFP fusion proteins using Western blotting analysis.

### 2.6 Luciferase activity assays

To understand the association of large yellow croaker SARM and TRIF, TRAF3, IRF3, or IRF7, HEK 293T cells in 24-well plates (1 × 10^5^ cells/well) were transiently co-transfected with pNF-κB-luc (100 ng/well, Clontech, Palo Alto, CA), pGL4-IRF3-pro (100 ng/well, Chinese invention patent number: ZL201710457836.8), pGL4-IRF7-pro (100 ng/well, Chinese invention patent number: ZL201710457820.7), or pGL4-IFN1-pro (Chinese invention patent application number: 201710456729.3), and pRL-TK (10 ng/well, Promega, Madison, WI) together with 100 ng pcDNA3.1-SARM and pcDNA3.1-TRIF, pcDNA3.1-TRAF3, pcDNA3.1-IRF3, or pcDNA3.1-IRF7 alone or in a combination of two using Lipofectamine 3000. The total amount of the transfected plasmids was then balanced by the pcDNA3.1 empty vector. At 24 hpt, the cells were then collected and lysed with passive lysis buffer (100 μL/well, Promega), with the centrifuged supernatant collected and used for luciferase reporter assay on a Promega GloMax^®^ 20/20 luminometer. The luciferase activity was calculated and normalized to the *Renilla* luciferase activity and presented as fold relative to the control group as described previously (28, 29).

### 2.7 Co-immunoprecipitation and western blotting assays

For co-immunoprecipitation experiments, HEK 293T cells in 6-well plates (1 × 10^6^ cells per well) were co-transfected with 2.5 μg of p3xFLAG-SARM together with 2.5 μg of pTurbo-TRIF-GFP, pTurbo-TRAF3-GFP, pTurbo-IRF3-GFP, pTurbo-IRF7-GFP, or pTurboGFP-N (control) in a combination of any two. At 24 hpt, the cells were collected and lysed with 500 μL RIPA buffer (Beyotime, Shanghai, China), with the cellular debris removed by centrifugation at 12000 g for 5 min at 4°C. After that, the cell supernatants were incubated with the Anti-Flag resin conjugated agarose beads (ANTI-FLAG^®^ M2 Affinity Gel, Sigma) overnight at 4°C, followed by 6 times wash with ice-cold PBS. The proteins bound to the agarose beads were eluted with 20 μL 5 × SDS sample buffer by boiling for 20 min at 100°C, and the precipitates were examined by Western blotting analysis with Anti-Flag and Anti-TurboGFP antibodies.

Western blotting analysis was performed as per our previous reports (28, 29). Briefly, the proteins were separated by 10% SDS-PAGE gels and then transferred to polyvinylidene difluoride (PVDF) membrane with pore size of 0.45 μm (Millipore Corporation). The transferred membrane was blocked with 5% non-fat dry milk in PBS for 1 h at room temperature. After one wash with PBS containing 0.1% Tween-20 (PBST) and two washes with PBS, each for 5 min, the membrane was incubated with rabbit polyclonal Anti-TurboGFP antibody or mouse monoclonal Anti-Flag antibody (diluted with antibody diluent at 1:5000) at 4°C overnight. After that, the membrane was washed three times as described above, and then incubated with the secondary antibody (diluted with antibody diluent at 1:2000) for 1 h at room temperature, followed by washing for three times. Subsequently, the bands were visualized using WesternBright™ ECL HRP substrate (Advansta, San Jose, USA) and ECL Western blotting system (LAS-4000mini, Fujifilm, Tokyo, Japan) according to the manufacturers’ instructions.

### 2.8 Antiviral activity assays

For antiviral activity assays, EPC cells seeded in 6-well plates (2 × 10^6^ cells per well) overnight were co-transfected with 2.5 μg pcDNA3.1-SARM together with 2.5 μg pcDNA3.1-TRIF, pcDNA3.1-TRAF3, pcDNA3.1-IRF3, or pcDNA3.1-IRF7 alone or in a combination of two. The pcDNA3.1 empty vector was added to balance the total amount of the transfected plasmids. At 24 hpt, the transfected cells were washed and infected with SVCV at an MOI of 1, with the cells collected for total RNA extraction at 24 h post-infection, followed by subsequent mRNA expression pattern evaluation of SVCV Glycoprotein (*SVCV-G*) and Matrix protein (*SVCV-M*) by qRT-PCR analysis. The expression data was calculated using the comparative Ct method (2^−ΔΔCt^) as described above, with the viral genes normalized to the *β-actin* of EPC. The primers used for qRT-PCR analysis are shown in **Table 1**.

### 2.9 Effects of SARM with TRIF, TRAF3, IRF3, and IRF7 on immune-related genes expression

To reveal the impacts of large yellow croaker SARM with TRIF, TRAF3, IRF3, and IRF7 on the expression profiles of the downstream immune-related genes of the signaling cascades, LYCMS cells cultured on 6-well plates with 4 × 10^5^ cells per well were co-transfected with 2.5 μg of pcDNA3.1-SARM together with 2.5 μg of pcDNA3.1-TRIF, pcDNA3.1-TRAF3, pcDNA3.1-IRF3, or pcDNA3.1-IRF7 alone or in a combination of two. The pcDNA3.1 empty vector was added to balance the total amount of the transfected plasmids. At 48 hpt, the transfected cells were then collected to extract total RNA for evaluating the mRNA expression patterns of the immune-related genes such as *IFN1*, *IRF3*, *IRF7*, *Mx*, *RSAD2*, and *ISG56* by qRT-PCR analysis. The primers used for qRT-PCR analysis are shown in **Table 1**.

### 2.10 Statistical analysis

All of the data obtained from qRT-PCR analysis and dual-luciferase reporter assays were presented as mean of three repeated experiments, with the bars representing the standard error (SE). Statistical analysis was performed using one-way analysis of variance (ANOVA), subsequent Duncan’s multiple range test was then carried out on SPSS version 20. The different superscripts above the bars indicate statistically different (*P* < 0.05), * *P* < 0.05, and ** *P* < 0.01 are considered statistically significant and remarkably significant, respectively.

## 3 Results

### 3.1 Sequence analysis of *Lc*-SARM

The full-length ORF of *Lc-SARM* was cloned from the gill of large yellow croakers, which consists of 2,154 bp and encodes a protein of 717 amino acids (aa) (GenBank accession number: OP225399). Three conserved domains were found in *Lc-*SARM based on CDD and SMART analysis, including an N-terminal ARM domain (67–317 aa), two SAM domains (411–479 and 480–547 aa), and a C-terminal TIR domain (560–697 aa) (**Supplementary Figure 1**). Multiple alignment analysis of *Lc*-SARM with other vertebrate SARM orthologs, including that from mandarin fish, medaka (*Oryzias latipes*), zebrafish, grass carp, rainbow trout (*Oncorhynchus mykiss*), African clawed frog (*Xenopus tropicalis*), chicken (*Gallus gallus*), mouse (*Mus musculus*), and human (*Homo sapiens*) showed that SARM was a conserved molecule in vertebrates (**Supplementary Figure 1**). The amino acid sequence of *Lc*-SARM has a similarity of 94% with mandarin fish, 91% with medaka, 90% with zebrafish, 90% with grass carp, and 89% with rainbow trout, which presented a strong homology among different fish. Moreover, it also exhibited a high amino acid sequence similarity with that from similarity with that from amphibian (77% with African clawed frog), bird (77% with chicken), and mammals (76% with human and 75% with mouse) (**Table 2**).

**Table 2:** Amino acid identity and similarity among *Lc-*SARM and SARM from other vertebrates.

Common name	Scientific name	Accession No.	Length (aa)	Identity	Similarity
Mandarin fish	*Siniperca chuatsi*	QWW30847.1	712	92%	94%
Medaka	*Oryzias latipes*	XP_004075485.1	713	84%	91%
Zebrafish	*Danio rerio*	NP_001124068.1	713	82%	90%
Grass carp	*Ctenopharyngodon idella*	AGM20429.2	713	82%	90%
Rainbow trout	*Oncorhynchus mykiss*	XP_036821319.1	724	84%	89%
African clawed frog	*Xenopus tropicalis*	XP_002937189.2	719	61%	77%
Chicken	*Gallus gallus*	XP_415814.5	717	61%	77%
Human	*Homo sapiens*	NP_055892.2	724	61%	76%
Mouse	*Mus musculus*	NP_766383.2	724	61%	75%

**Figure 1:**
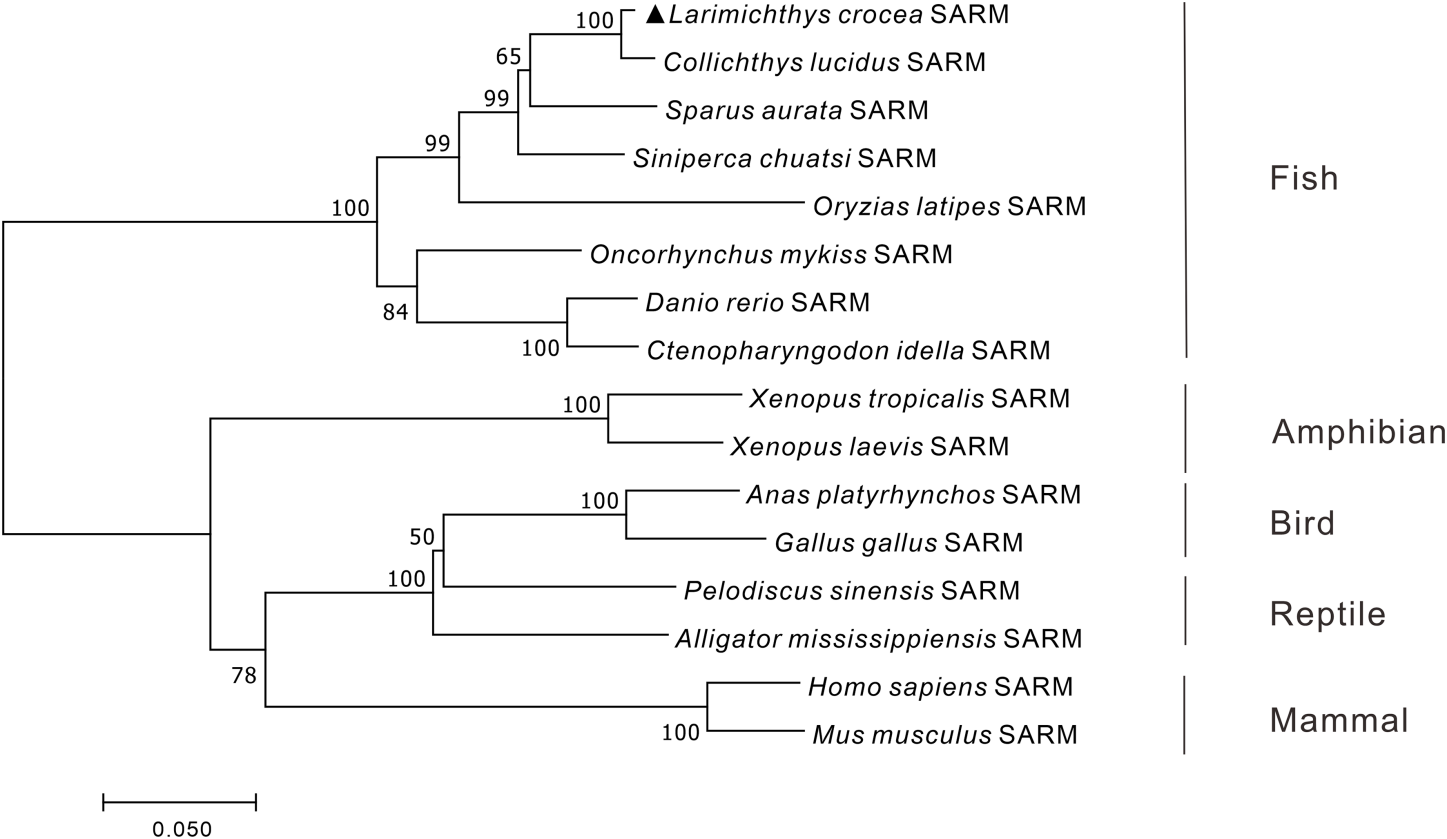
Phylogenetic analysis of vertebrate SARM. The phylogenetic tree was constructed base on the protein sequences of vertebrate SARM by using the neighbor-joining method within the MEGA version 7.0 software, with 10,000 replications of the bootstrap test. The GenBank association numbers of the SARM orthologues are shown as follows: *L. crocea*, OP225399; *C. lucidus*, TKS81088.1; *Sparus aurata*, XP_030249993.1; *S. chuatsi*, QWW30847.1; *O. latipes*, XP_004075485.1; *O. mykiss*, XP_036821319.1; *D. rerio*, NP_001124068.1; *C. idella*, AGM20429.2; *X. tropicalis*, XP_002937189.2; *X. laevis*, XP_018101625.1; *Anas platyrhynchos*, XP_038021639.1; *G. gallus*, XP_415814.5; *Pelodiscus sinensis*, XP_006118078.1; *Alligator mississippiensis*, KYO40867.1; *H. sapiens*, NP_055892.2; *M. musculus*, NP_766383.2.

To reveal the phylogenic relationship of SARM in vertebrates, the phylogenetic tree of vertebrate SARM orthologs was constructed by using the neighbor-joining method with 10,000 replications in the bootstrap test in MEGA 7.0 program. The results showed that the amphibian, bird, reptile, and mammalian SARM orthologs were clustered into one branch, whereas the teleost SARM orthologs were clustered into another branch, with *Lc*-SARM firstly clustered to that in spinyhead croaker (*Collichthys lucidus*) (**Figure 1**).

### 3.2 Genomic organization of *SARM* genes in vertebrates

**Figure 2:**
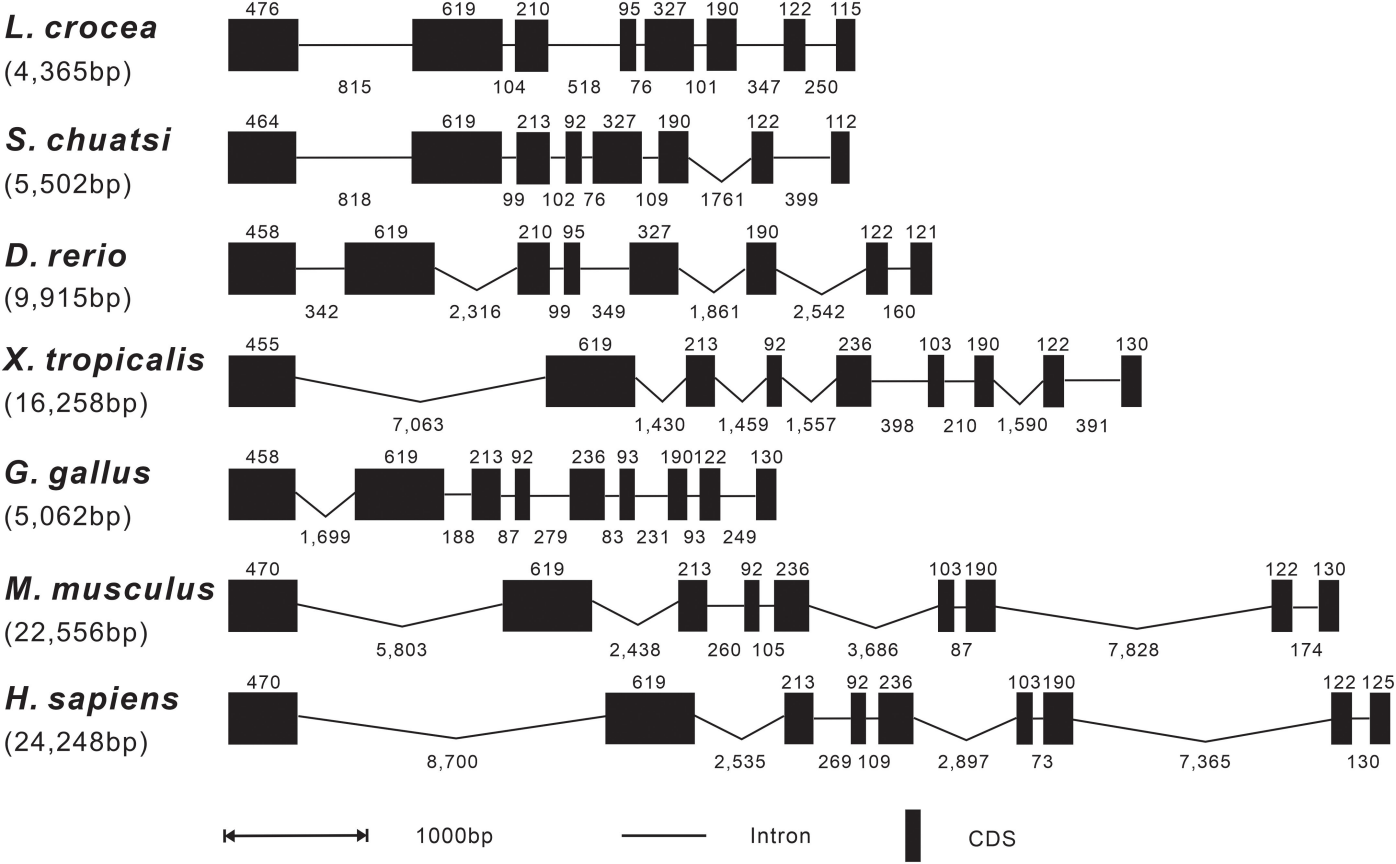
Genomic organization comparison of *Lc-SARM* with other vertebrates. Comparison of the genomic organization of *SARM* gene in *S. chuatsi*, *D. rerio*, *X. tropicalis*, *G. gallus*, *M. musculus*, and *H. sapiens*. Exons are represented by black boxes, with the lengths in base pairs (bp) shown above the black boxes. Introns are represented by black lines, and the lengths in bp are shown below the lines. Gene sequence information and the GenBank accession numbers are presented as follows: *L. crocea*, NC_040017.1 (14896355-14890040); *S. chuatsi*, NC_058048.1 (16261494-16255179); *D. rerio*, NC_007126.7 (28199762-28189552); *X. tropicalis*, NC_030678.2 (34935414-34914211); *G. gallus*, NC_052550.1 (6133119-6139992); *M. musculus*, NC_000077.7 (78388642-78361099); and *H. sapiens*, NC_000017.11 (28368458-28407285).

The genomic organization of *Lc-SARM* was analyzed and compared to that from teleosts to mammals, including mandarin fish, zebrafish, African clawed frog, chicken, mouse, and human. The result showed that the genomic sequences of *SARM* had a range from 4,365 bp to 24,248 bp in length of the detected species, with the shortest discovered in large yellow croaker and the longest found in human (**Figure 2**). The exon-intron organization of *SARM* in large yellow croaker was constituted of 8 exons and 7 introns, consistent with that found in other teleosts, including mandarin fish and zebrafish. However, it was varied from that found in amphibian, bird, and mammals, which was constituted of 9 exons and 8 introns instead. In addition, the size of the exons was conserved between large yellow croaker and zebrafish, with some differences in the first and the last exons, which was 476 bp and 115 bp in the first and the last exon of large yellow croaker, whereas 458 bp and 121 bp of zebrafish. Notably, the third and the fourth exon in mandarin fish showed some distinction from that found in large yellow croaker and zebrafish, with 210 bp and 95 bp in the third and fourth exon of large yellow croaker and zebrafish, whereas 213 bp and 92 bp in mandarin fish (**Figure 2**).

### 3.3 Subcellular localization of *Lc*-SARM

**Figure 3:**
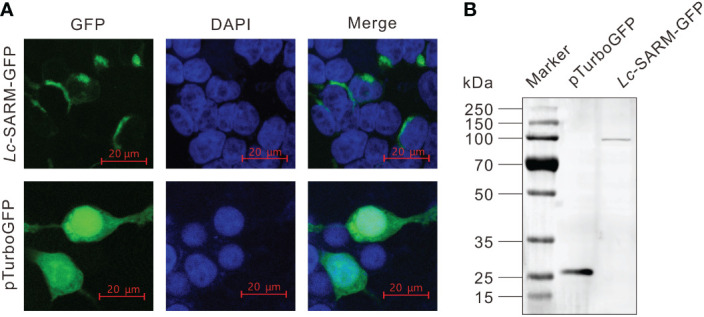
Subcellular localization of *Lc-*SARM. **(A)** HEK 293T cells were transfected with pTurbo-SARM-GFP and pTurboGFP-N (vector control), respectively. At 24 hpt, the cells were stained with DAPI and then detected and photographed under a confocal microscope. **(B)** The confirmation of the expression of *Lc*- SARM-GFP and pTurboGFP fusion proteins was conducted by Western blotting analysis using the Anti-TurboGFP antibody.

To determine the subcellular localization of large yellow croaker SARM, the full-length ORF of *Lc-*SARM was cloned and inserted into the pTurboGFP-N expression vector for transient transfection into HEK 293T cells, and then examined under a confocal microscope. It was revealed that the *Lc*-SARM-GFP fusion protein was distributed in the cytoplasm. In contrast, the GFP fusion protein in the control cells that transfected with the empty pTurboGFP-N vector presented a cytosolic as well as nucleic distribution (**Figure 3A**). Subsequent Western blotting analysis with the Anti-TurboGFP antibody revealed that the molecular weight of *Lc*-SARM-GFP fusion protein was near 100 kDa, whereas the pTurboGFP (control) was over 25 kDa (**Figure 3B**). The calculated weight of *Lc*-SARM was about 80.6 kDa, with the addition of the pTurboGFP, it is therefore confirmed the successful expression of *Lc*-SARM-GFP fusion protein in HEK 293T cells.

### 3.4 Constitutive and inductive expression patterns of *Lc-SARM*

**Figure 4:**
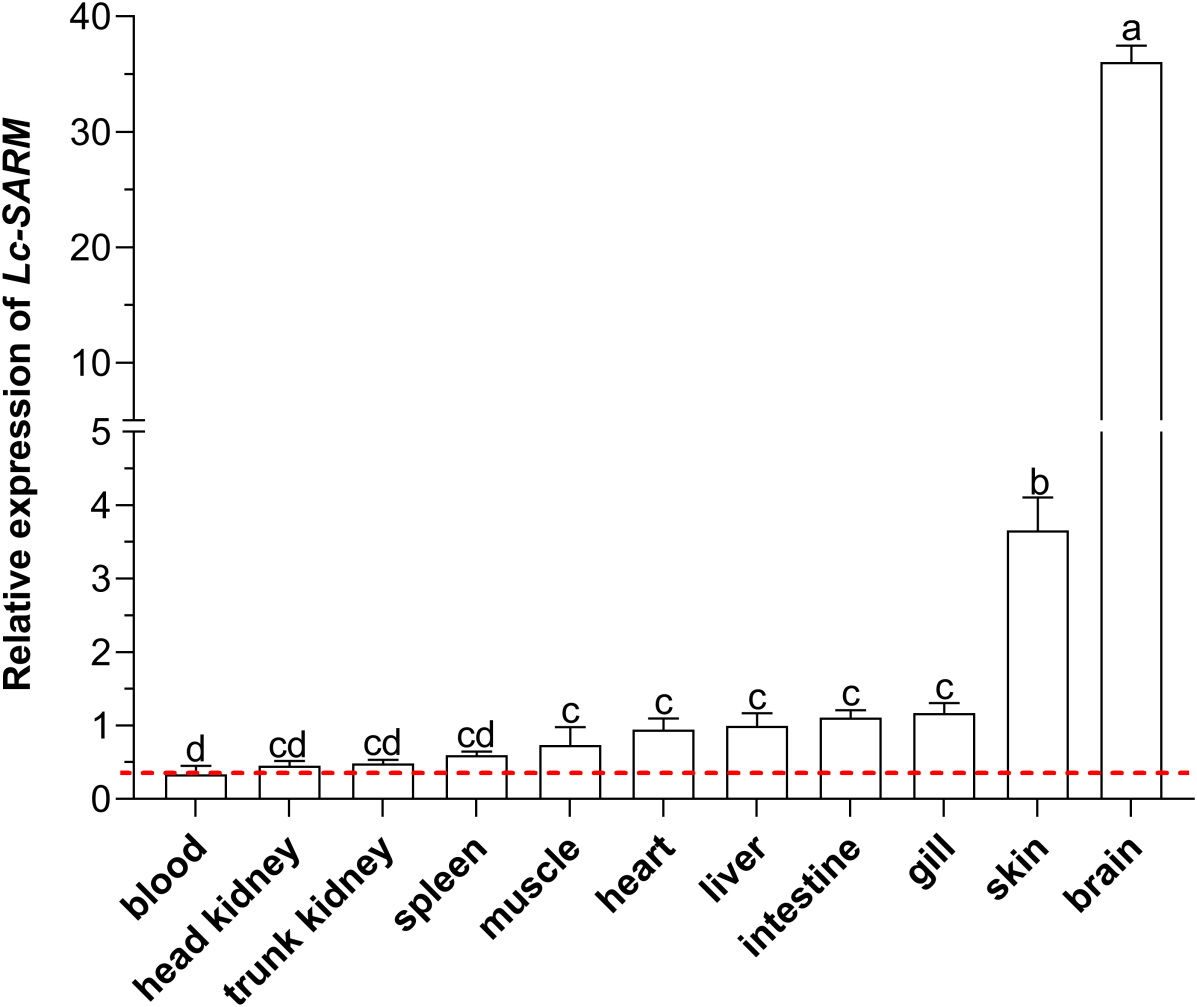
Organs/tissues distribution analysis of *Lc-SARM* in large yellow croaker. The mRNA expression levels of *Lc-SARM* in 11 various organs/tissues of the healthy fish (n = 6) were detected by qRT-PCR analysis, with normalizing to the expression of *L. crocea β-actin*. The lowest expression level of *Lc-SARM* detected in the blood was marked with a red dotted base line. All of the data are shown as the mean of three individual experiments, with bars representing the SE. Different letters above the bars indicate statistically significant differences between the groups (*P* < 0.05).

To obtain the expression patterns of *Lc-SARM* in different organs/tissues of normal large yellow croakers, the blood, head kidney, spleen, trunk kidney, muscle, heart, intestine, liver, gill, skin, and brain from healthy fish were collected for mRNA expression analysis. The results of qRT-PCR revealed that the expression profiles of *Lc-SARM* were generally found in all the detected organs/tissues, with the highest expression level observed in the brain, followed by the skin, gill, intestine, liver, heart, muscle, spleen, trunk kidney, and head kidney (**Figure 4**).

**Figure 5:**
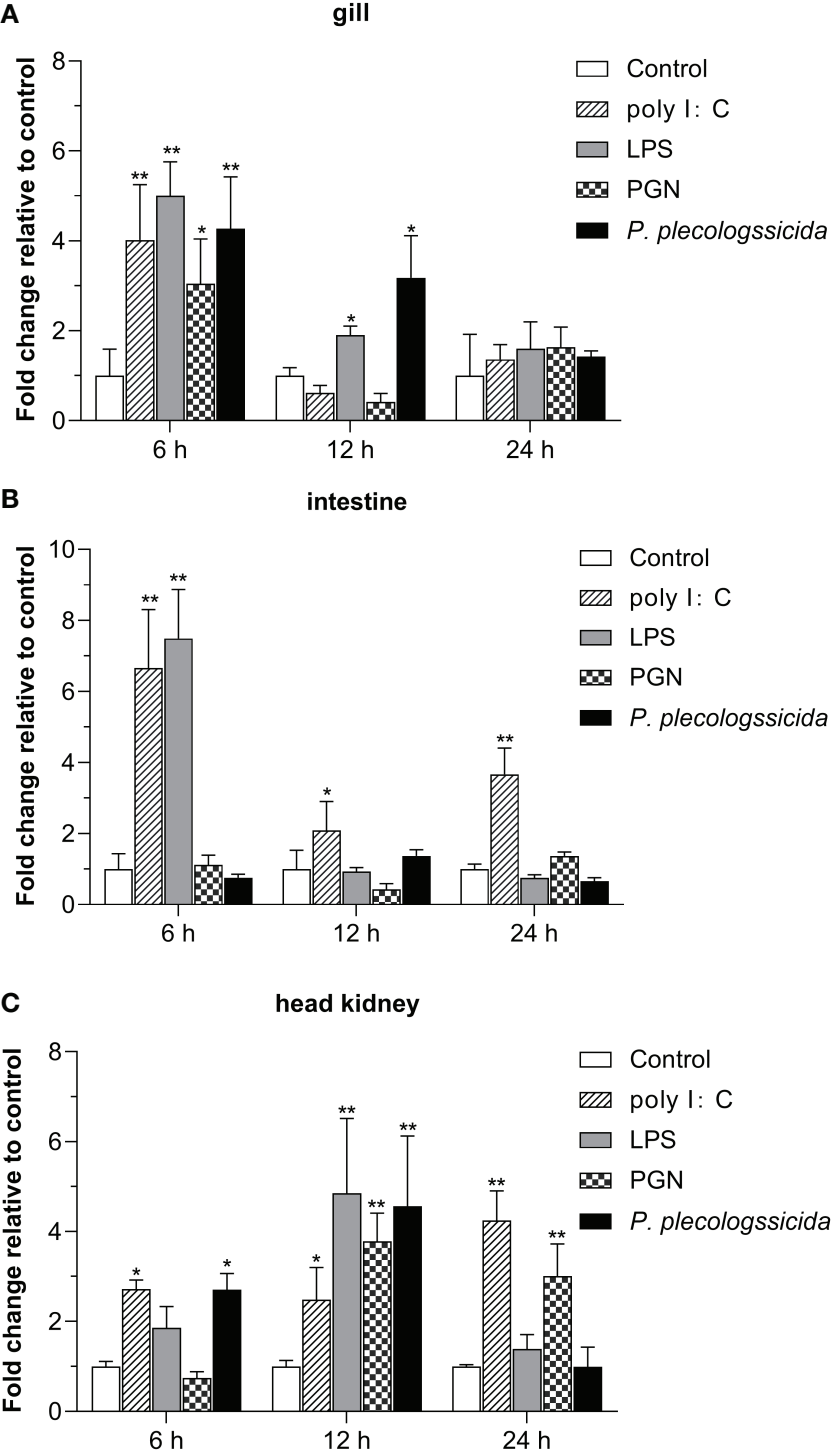
Expression patterns of *Lc-SARM* in response to the stimulations of poly I:C, LPS, PGN, and *P. plecoglossicida*. The healthy large yellow croakers (five groups) were injected intraperitoneally with 100 μL of poly I:C (1 mg/mL), LPS (0.5 mg/mL), PGN (1 mg/mL), *P. plecoglossicida* suspension in PBS (5 × 10^5^ CFU/mL), and sterile PBS (control), respectively. Six large yellow croakers were randomly selected from each group at 6, 12, and 24 hpi and the mRNA expression levels of *Lc-SARM* in the gill **(A)**, intestine **(B)**, and head kidney **(C)** were examined by qRT-PCR. The results were calculated by normalization to the expression of *L. crocea β-actin* and then recorded as fold change relative to the PBS injection group (control) at the same time point. All of the data are shown as the mean of three individual experiments, with bars representing the SE. Statistically significant differences are indicated by the asterisks (* *P* < 0.05, ** *P* < 0.01).

To further investigate the function of *Lc-*SARM in the host immune responses, the mRNA expression profiles of *Lc-SARM* gene were examined in the mucosal immune tissues (gill and intestine) and the peripheral immune organ (head kidney) in response to the stimulations of poly I:C, LPS, PGN, and also the infection of *P. plecoglossicida*. The results showed that, in comparison with the control group, *Lc-SARM* was dramatically induced upon poly I:C challenge in the gill, intestine, and head kidney, with a 4.0-fold increase at 6 hpi in the gill (**Figure 5A**), a 6.7-, 2.1-, and 3.7-fold increase at 6, 12, and 24 hpi in the intestine (**Figure 5B**), a 2.7-, 2.4-, and 4.2-fold increase at 6, 12, and 24 hpi in the head kidney (**Figure 5C**). The expression profile of *Lc-SARM* was also significantly up-regulated in response to the LPS challenge, with a 5.0- and 1.9-fold increase at 6 and 12 hpi in the gill (**Figure 5A**), a 7.5-fold increase at 6 hpi in the intestine (**Figure 5B**), a 4.8-fold increase at 12 hpi in the head kidney (**Figure 5C**), respectively. In addition, *Lc-SARM* was significantly up-regulated under PGN stimulation, with a 3.0-fold increase at 6 hpi in the gill (**Figure 5A**), a 3.8- and 3.0-fold increase at 12 and 24 hpi in the head kidney (**Figure 5C**), respectively. Additionally, *Lc-SARM* was also significantly up-regulated in response to *P. plecoglossicida* infection, with a 4.3- and 3.2-fold increase at 6 and 12 hpi in the gill (**Figure 5A**), a 2.7- and 4.6-fold increase at 6 and 12 hpi in the head kidney (**Figure 5C**), respectively.

### 3.5 *Lc*-SARM suppresses TRIF and TRAF3 mediated NF-κB promoter activation

**Figure 6:**
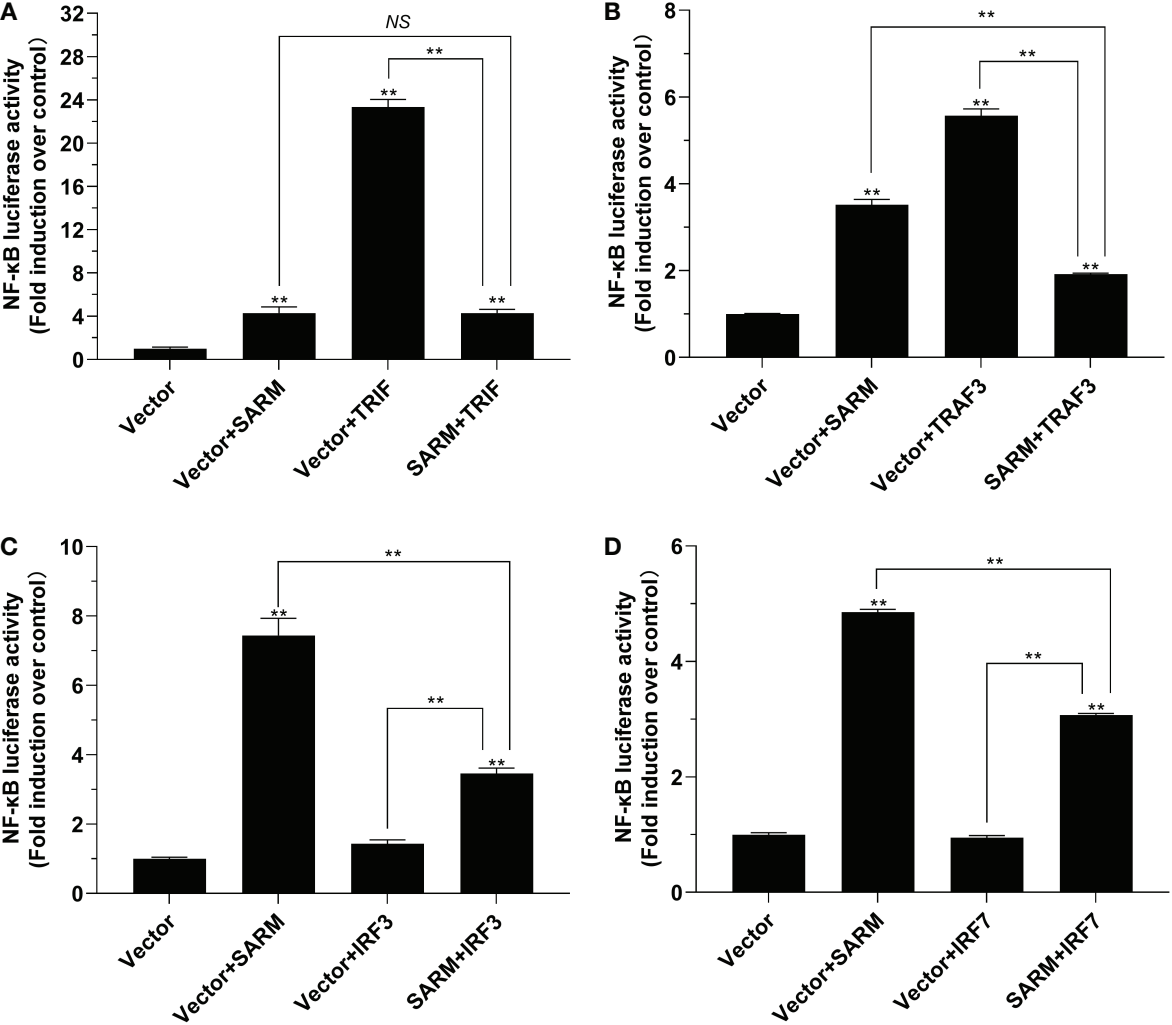
The role of *Lc-*SARM in the regulation of NF-κB signaling. The associations of *Lc-*SARM with *Lc-*TRIF **(A)**, *Lc-*TRAF3 **(B)**, *Lc-*IRF3 **(C)**, and *Lc-*IRF7 **(D)** in the NF-κB promoter activation were analyzed by luciferase reporter assays. HEK 293T cells seeded in 24-well plates (1 × 10^5^ cells/well) were co-transfected with 100 ng of pNF-κB-luc and 10 ng of pRL-TK together with 100 ng of pcDNA3.1-SARM, pcDNA3.1-TRIF, pcDNA3.1-TRAF3, pcDNA3.1-IRF3, and pcDNA3.1-IRF7 alone or in a combination of two. For each transfection, the amounts of transfected plasmids were balanced with the pcDNA3.1 empty vector. At 24 hpt, the cells were collected and lysed for the measurement of luciferase activities. All data are shown as mean of three independent experiments, with bars representing the SE. *P < 0.05; **P < 0.01; NS, not significant.

The results of dual-luciferase reporter assays showed that *Lc*-SARM overexpression could significantly induce NF-κB promoter activation. However, the co-transfection of *Lc*-SARM with *Lc*-TRIF significantly impaired the activation of NF-κB promoter in comparison with the *Lc*-TRIF alone (**Figure 6A**). Moreover, the co-transfection of *Lc*-SARM with *Lc*-TRAF3 significantly reduced the induction of NF-κB promoter activity in comparison with the transfection of *Lc*-SARM or *Lc*-TRAF alone (**Figure 6B**). In addition, the co-transfection of *Lc*-SARM with *Lc*-IRF3 or *Lc*-IRF7 significantly suppressed the induction of NF-κB promoter activity compared to the only overexpression of *Lc*-SARM, although *Lc*-IRF3 or *Lc*-IRF7 overexpression showed little effect on the activation of NF-κB promoter activity (**Figures 6C, D**).

### 3.6 *Lc*-SARM suppresses TRIF, TRAF3, and IRF3/7 mediated IRF3 and IRF7 promoter activation

**Figure 7:**
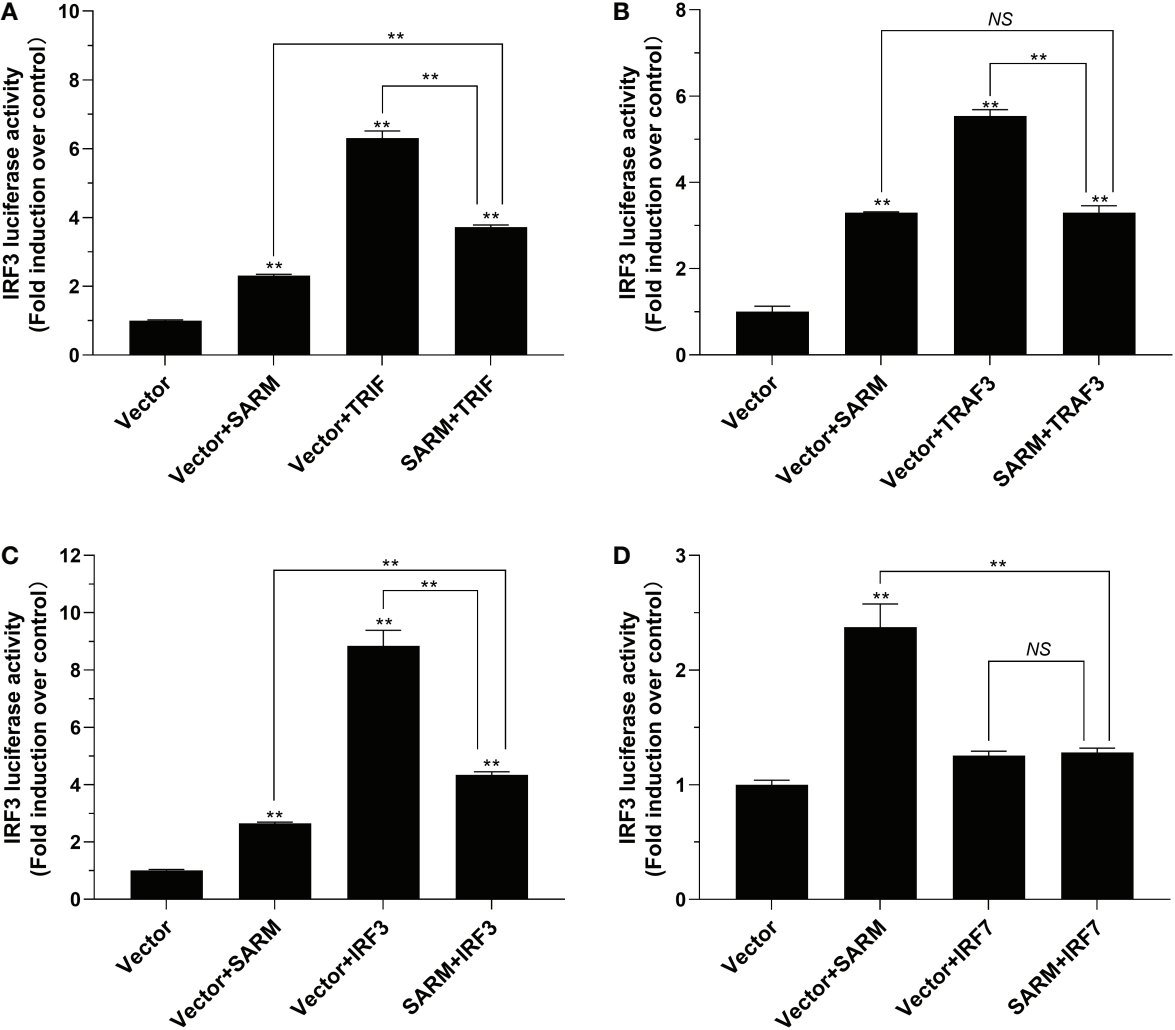
The role of *Lc-*SARM in the regulation of IRF3 signaling. The associations of *Lc-*SARM with *Lc-*TRIF **(A)**, *Lc-*TRAF3 **(B)**, *Lc-*IRF3 **(C)** and *Lc-*IRF7 **(D)** in the IRF3 promoter activation were analyzed by luciferase reporter assays. HEK 293T cells seeded in 24-well plates (1 × 10^5^ cells/well) were co-transfected with 100 ng of pGL4-IRF3-pro and 10 ng of pRL-TK together with 100 ng of pcDNA3.1-SARM, pcDNA3.1-TRIF, pcDNA3.1-TRAF3, pcDNA3.1-IRF3, and pcDNA3.1-IRF7 alone or in a combination of two as described above. The cells were harvested at 24 hpt and used for the measurement of luciferase activities. All data are expressed as mean of three independent experiments, with bars representing the SE. *P < 0.05; **P < 0.01; NS, not significant.

**Figure 8:**
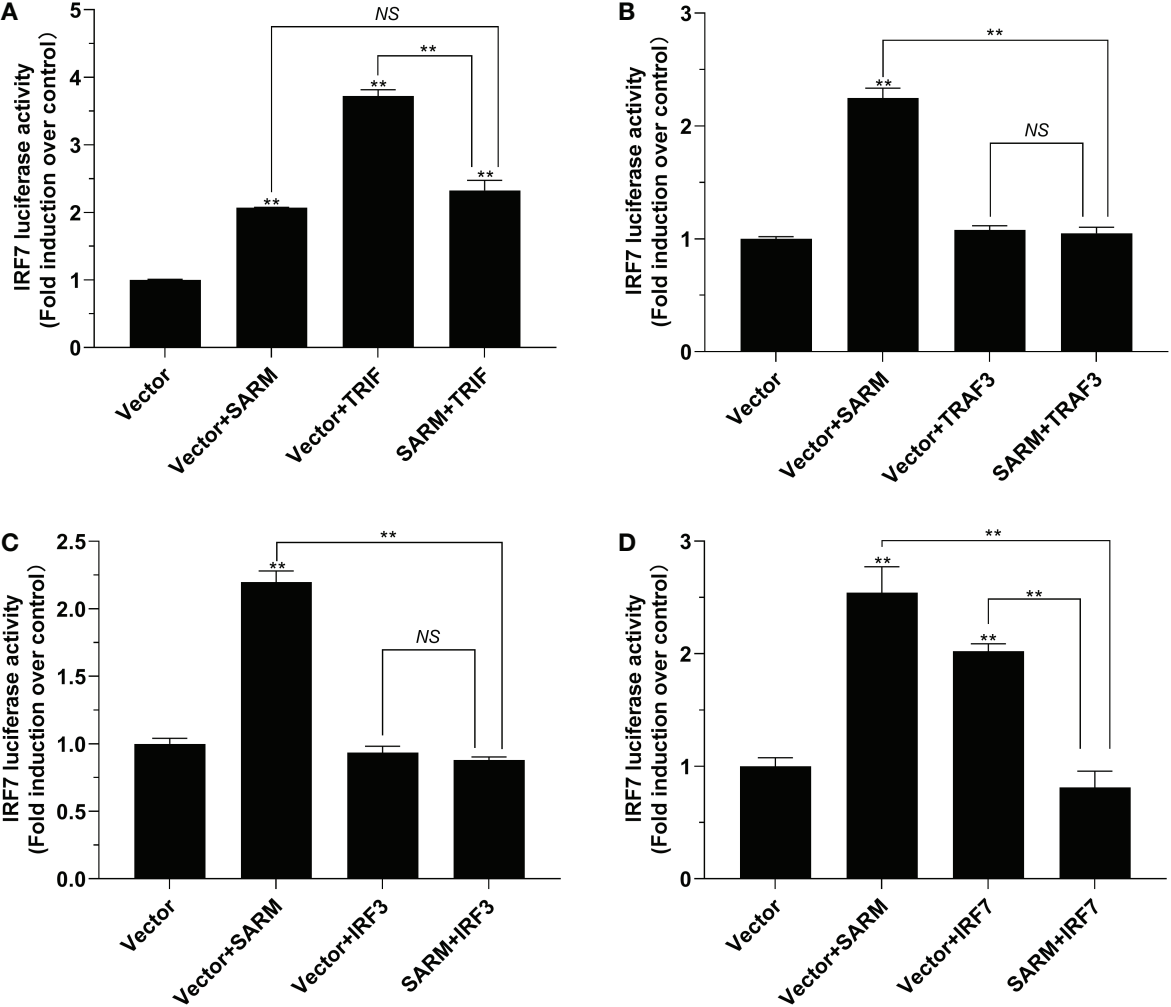
The role of *Lc-*SARM in the regulation of IRF7 signaling. The associations of *Lc-*SARM with *Lc-*TRIF **(A)**, *Lc-*TRAF3 **(B)**, *Lc-*IRF3 **(C)** and *Lc-*IRF7 **(D)** in the IRF7 promoter activation were analyzed by luciferase reporter assays. HEK 293T cells seeded in 24-well plates were co-transfected with 100 ng of pGL4-IRF7-pro and 10 ng pRL-TK together with 100 ng of pcDNA3.1-SARM, pcDNA3.1-TRIF, pcDNA3.1-TRAF3, pcDNA3.1-IRF3, and pcDNA3.1-IRF7 alone or in a combination of two as described above. The cells were harvested at 24 hpt and used for the measurement of luciferase activities. All data are presented as mean of three independent experiments, with bars representing the SE. *P < 0.05; **P < 0.01; NS, not significant.

To investigate the role of *Lc*-SARM in IRF3 and IRF7 signaling, the dual-luciferase reporter assays were also performed to detect the effect of *Lc*-SARM on IRF3 and IRF7 promoter activation. The results showed clearly that *Lc*-SARM overexpression could significantly induce IRF3 and IRF7 promoter activation, however, co-transfection of *Lc*-SARM with *Lc*-TRIF, *Lc*-TRAF3, or *Lc*-IRF3 significantly abolished the induction level of IRF3 promoter activity in contrast to the only overexpression of *Lc*-TRIF, *Lc*-TRAF3, or *Lc*-IRF3 (**Figures 7A-C**), respectively. In addition, the co-transfection of *Lc*-SARM with *Lc*-TRIF or *Lc*-IRF7 also significantly suppress the induction of IRF7 promoter activity compared to the only overexpression of *Lc*-TRIF or *Lc*-IRF7 (**Figures 8A, D**). Notably, although overexpression *Lc*-IRF7 showed little effect on the activation of IRF3 promoter (**Figure 7D**), *Lc*-TRAF3 and *Lc*-IRF3 exhibited no significant function on the induction of IRF7 promoter activity (**Figures 8B, C**), the co-expression of *Lc*-SARM with those molecules significantly abolished *Lc*-SARM mediated IRF3 or IRF7 promoter activation (**Figures 7D**, **8B, C**).

### 3.7 *Lc*-SARM suppresses TRIF and IRF3 mediated type I IFN promoter activation

**Figure 9:**
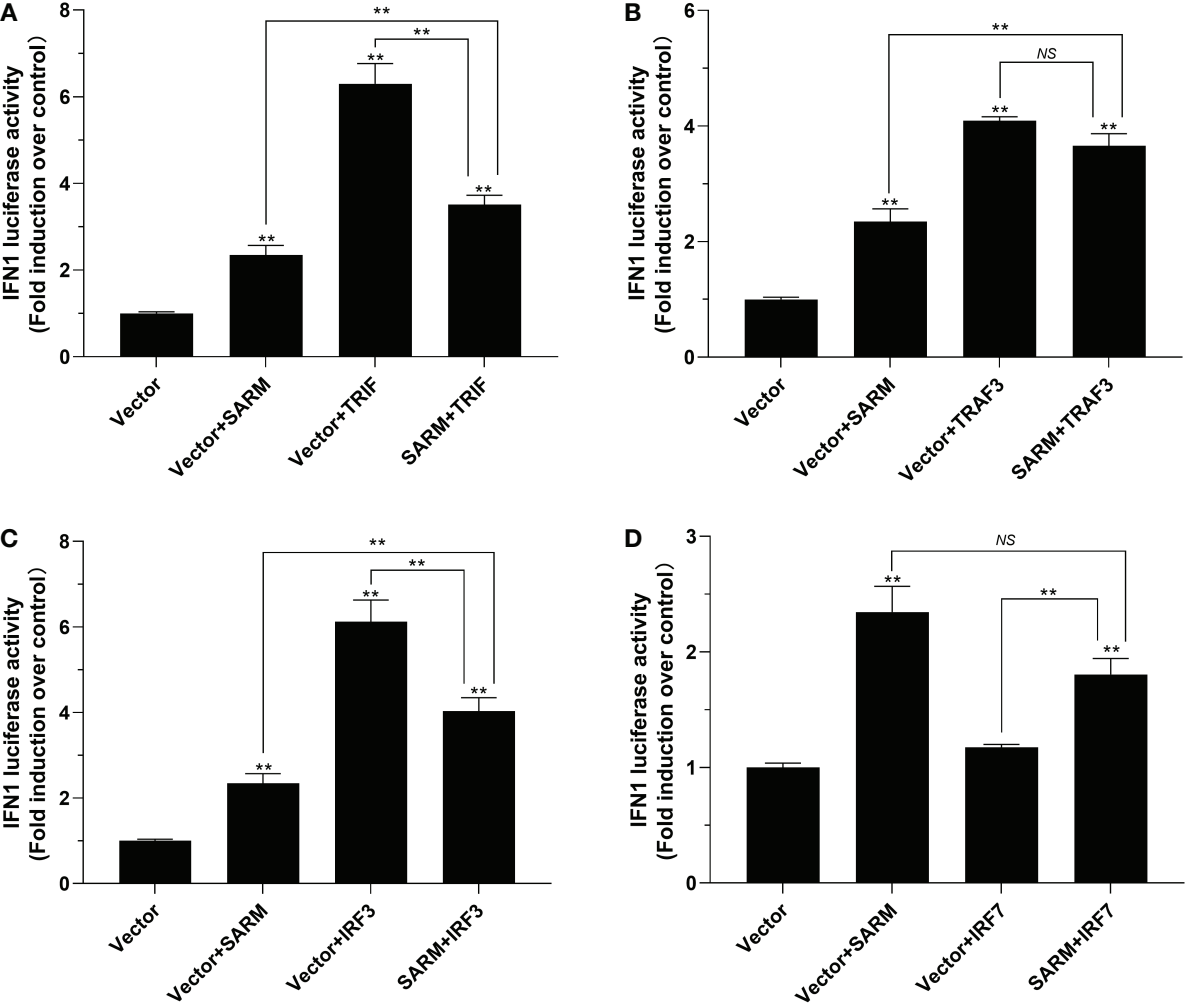
The role of *Lc-*SARM in the regulation of type I IFN signaling. The associations of *Lc-*SARM with *Lc-*TRIF **(A)**, *Lc-*TRAF3 **(B)**, *Lc-*IRF3 **(C)** and *Lc-*IRF7 **(D)** in the type I IFN promoter activation were analyzed by luciferase reporter assays. HEK 293T cells seeded in 24-well plates were co-transfected with 100 ng of pGL4-IFN1-pro and 10 ng pRL-TK together with 100 ng of pcDNA3.1-SARM, pcDNA3.1-TRIF, pcDNA3.1-TRAF3, pcDNA3.1-IRF3, and pcDNA3.1-IRF7 alone or in a combination of two as described above. At 24 h post transfection, the cells were harvested and used for the detection of luciferase activities. All data are presented as mean of three independent experiments, with bars representing the SE. *P < 0.05; **P < 0.01; NS, not significant.

To further determine the role of *Lc*-SARM in the activation type I IFN signaling, the dual-luciferase reporter assays were also conducted to detect the function of *Lc*-SARM on IFN1 promoter activation. The results showed that overexpression of *Lc*-SARM, *Lc*-TRIF, *Lc*-TRAF3, *Lc*-IRF3 alone could significantly induce IFN1 promoter activation, with *Lc*-IRF7 overexpression exhibited no significant effect (**Figures 9A-D**). However, co-transfection of *Lc*-SARM with *Lc*-TRIF or *Lc*-IRF3 could significantly suppress IFN1 promoter activation compared to the overexpression of *Lc*-TRIF or *Lc*-IRF3 alone (**Figures 9A, C**). Nevertheless, co-transfection of *Lc*-SARM with *Lc*-TRAF3 or *Lc*-IRF7 had no significant effect on the activation of IFN1 promoter activity compared to the only overexpression of *Lc*-TRAF3 or *Lc*-SARM (**Figures 9B, D**).

### 3.8 *Lc*-SARM interacts with TRIF, TRAF3, IRF3, and IRF7

**Figure 10:**
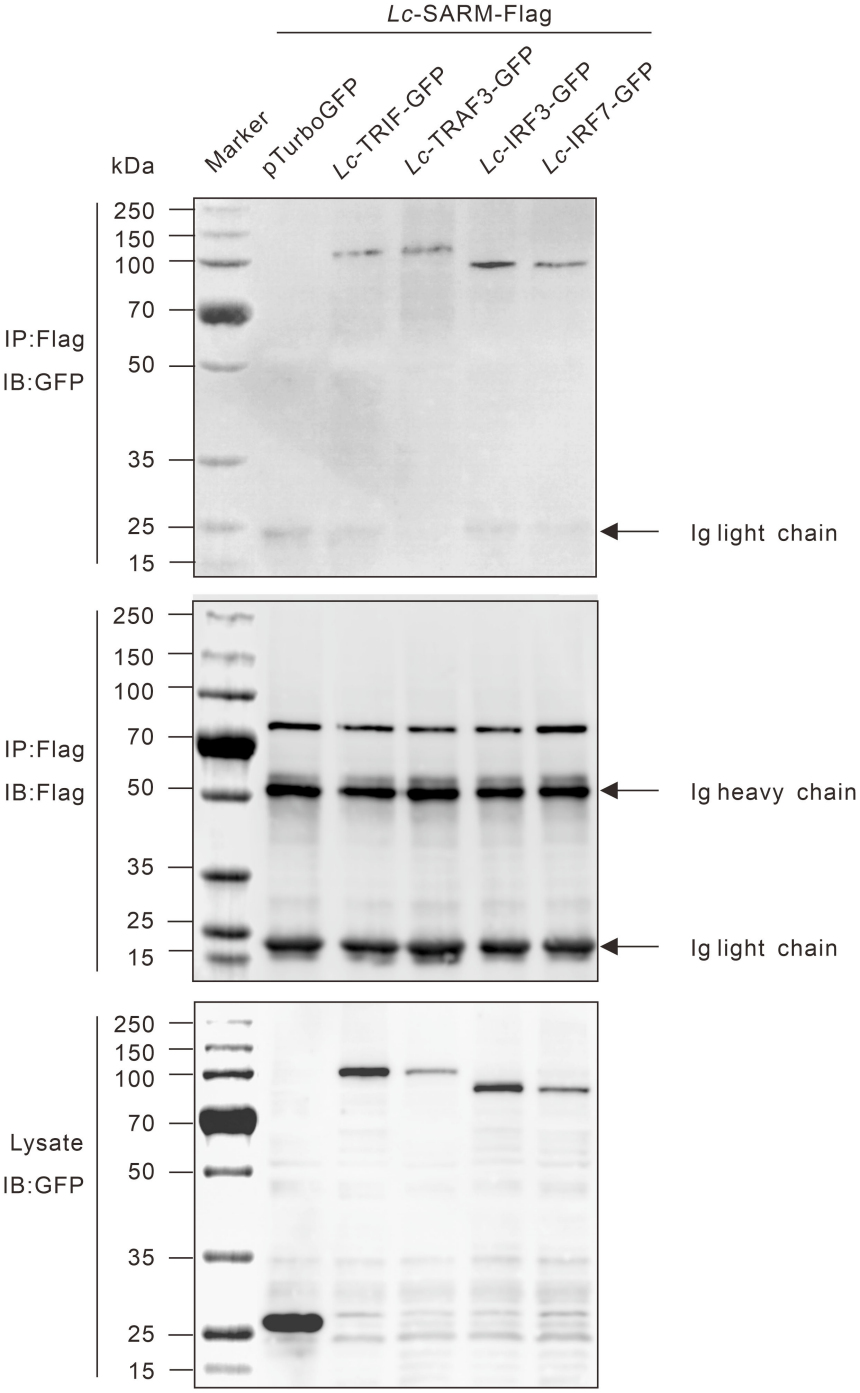
Interaction of *Lc*-SARM with *Lc*-TRIF, *Lc*-TRAF3, *Lc*-IRF3, and *Lc*-IRF7. HEK 293T cells seeded in 6-well plates (1 × 10^6^ cells/well) were co-transfected with 2.5 μg of p3xFLAG-SARM together with 2.5 μg of pTurbo-TRIF-GFP, pTurbo-TRAF3-GFP, pTurbo-IRF3-GFP, pTurbo-IRF7-GFP, or pTurboGFP-N (vector control) in a combination of two. At 24 hpt, the cells were collected and lysed, and the cell lysates were immunoprecipitated with Anti-Flag antibody (covalently conjugated to agarose beads) and immunoblotting with Anti-TurboGFP antibody (upper panels). The *Lc*-SARM-Flag bound to Anti-Flag-agarose beads was shown by immunoblotting with Anti-Flag antibody (middle panels), and the cells lysates were also detected by immunoblotting with Anti-TurboGFP antibody (bottom panels), respectively.

To determine the possible interaction of *Lc-*SARM with *Lc*-TRIF, *Lc*-TRAF3, *Lc*-IRF3, or *Lc*-IRF7, HEK 293T cells were co-transfected p3xFLAG-SARM along with pTurbo-TRIF-GFP, pTurbo-TRAF3-GFP, pTurbo-IRF3-GFP, pTurbo-IRF7-GFP, or pTurboGFP-N (control) in a combination of two, respectively. The results of co-immunoprecipitation assay revealed that Anti-Flag Ab-immunoprecipitated protein complexes, except the control cells transfected with p3xFLAG-SARM and pTurboGFP-N, were also recognized by Anti-TurboGFP antibody (**Figure 10**), indicating that *Lc*-SARM could interact with *Lc*-TRIF, *Lc*-TRAF3, *Lc*-IRF3, and *Lc*-IRF7.

### 3.9 *Lc*-SARM suppresses TRIF, TRAF3, IRF3, and IRF7 mediated antiviral signaling

**Figure 11:**
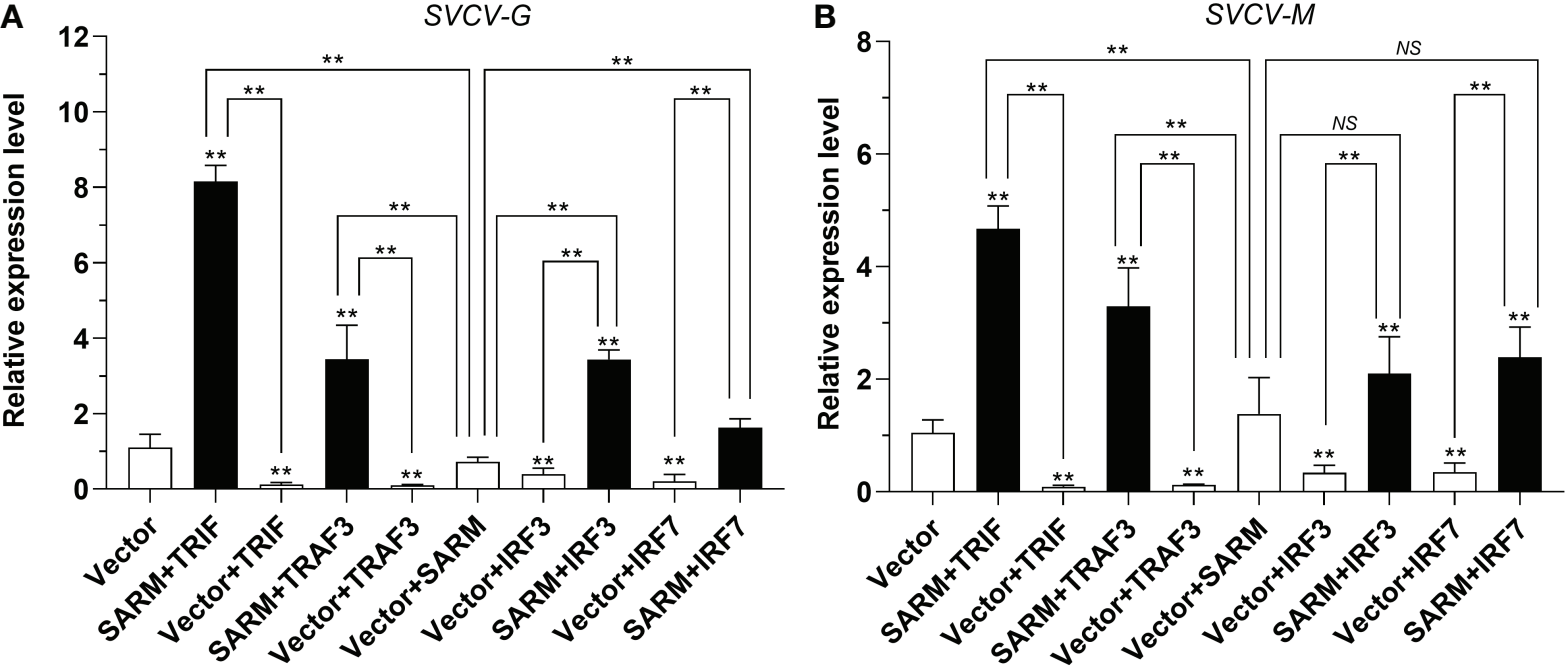
Antiviral effects of *Lc*-SARM co-expressed with *Lc*-TRIF, *Lc*-TRAF3, *Lc*-IRF3, and *Lc*-IRF7. EPC cells in 6-well plates (2 × 10^6^ cells/well) were co-transfected with 2.5 μg of pcDNA3.1-SARM and 2.5 μg of pcDNA3.1-TRIF, pcDNA3.1-TRAF3, pcDNA3.1-IRF3, or pcDNA3.1-IRF7 alone or in a combination of two, with the total amount of transfected plasmids balanced with the pcDNA3.1 empty vector. At 24 hpt, the cells were infected with SVCV at an MOI of 1, the cells were then collected at 24 h post-infection for total RNA extraction, and the mRNA expression levels of *SVCV-G* **(A)** and *SVCV-M* **(B)** were detected by qRT-PCR analysis, with normalizing to the expression of EPC *β-actin* using the 2^–ΔΔCt^ method. All data are obtained from three individual experiments, with bars representing the SE. *P < 0.05; **P < 0.01; NS, not significant.

To determine the association of *Lc*-SARM with *Lc*-TRIF, *Lc*-TRAF3, *Lc*-IRF3, and *Lc*-IRF7 in the host antiviral response, EPC cells were transfected with pcDNA3.1-SARM along with pcDNA3.1-TRIF, pcDNA3.1-TRAF3, pcDNA3.1-IRF3, or pcDNA3.1-IRF7 in a combination of two before SVCV infection. The results showed that the EPC cells only overexpression of *Lc*-TRIF, *Lc*-TRAF3, *Lc*-IRF3, or *Lc*-IRF7 could significantly decreased the mRNA expression levels of compared to the control cells transfected with the empty pcDNA3.1 vector at 24 h post-infection, whereas the only overexpression of *Lc*-SARM showed no obvious difference to that of the control cells on the expression of *SVCV-G* and *SVCV-M* (**Figures 11A, B**). Notably, co-expression of *Lc*-SARM with *Lc*-TRIF, *Lc*-TRAF3, *Lc*-IRF3, or *Lc*-IRF7 in EPC cells significantly increased the expression level of *SVCV-G* and *SVCV-M* compared to the overexpression of *Lc*-TRIF, *Lc*-TRAF3, *Lc*-IRF3, or *Lc*-IRF7 alone (**Figures 11A, B**). These results demonstrated that *Lc*-SARM can effectively abolish *Lc*-TRIF, *Lc*-TRAF3, *Lc*-IRF3, and *Lc*-IRF7 mediated antiviral response against SVCV infection.

**Figure 12:**
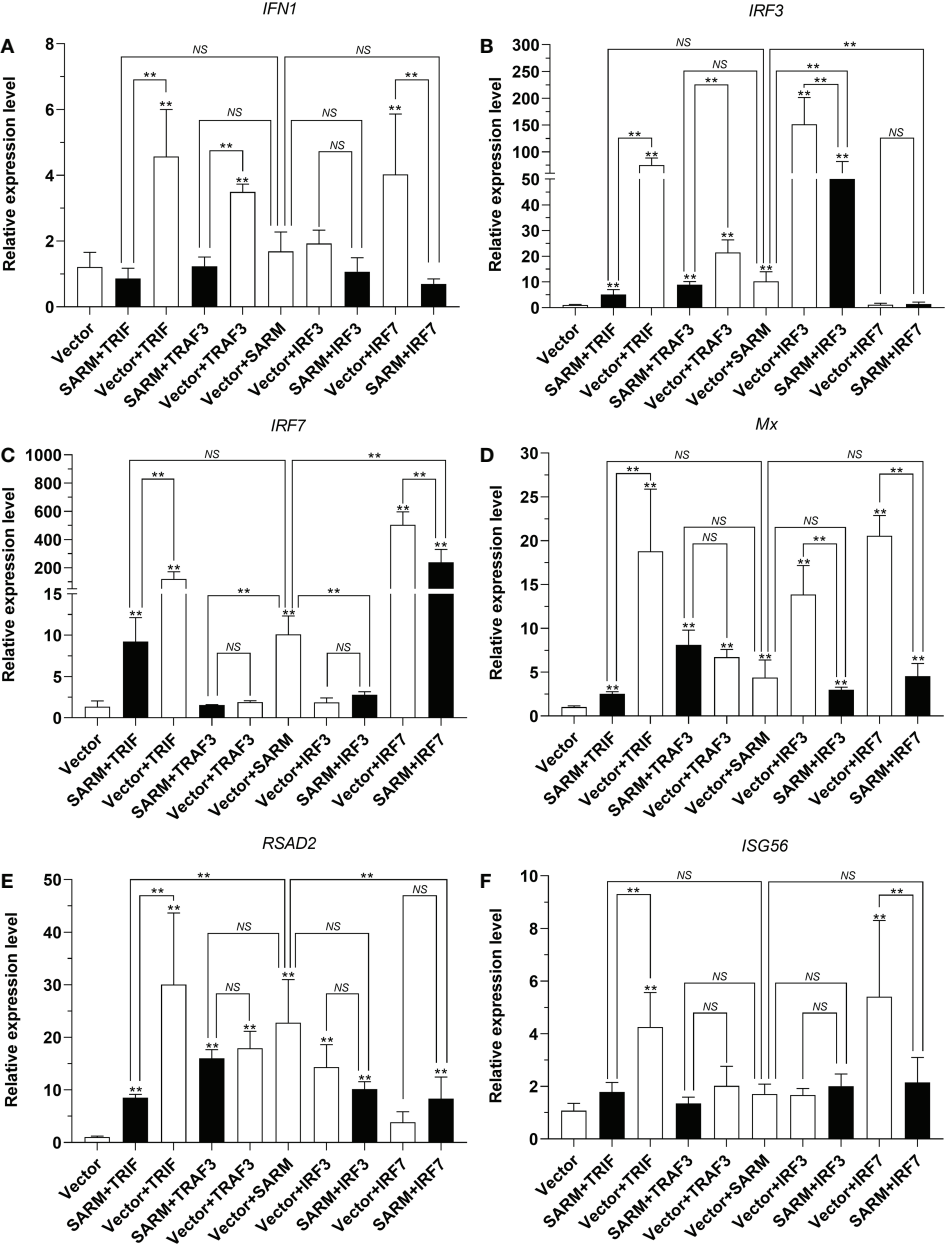
Association of *Lc*-SARM with *Lc*-TRIF, *Lc*-TRAF3, *Lc*-IRF3, and *Lc*-IRF7 in the induction of antiviral-related genes expression. LYCMS cells seeded on 6-well plates at a density of 4 × 10^5^ cells per well were co-transfected with 2.5 μg of pcDNA3.1-SARM together with 2.5 μg of pcDNA3.1-TRIF, pcDNA3.1-TRAF3, pcDNA3.1-IRF3, or pcDNA3.1-IRF7 alone or in a combination of two, with the total amount of transfected plasmids balanced with the pcDNA3.1 empty vector. At 48 hpt, the cells were collected to extract total RNA for evaluating the mRNA expression levels of antiviral-related genes including *IFN1* **(A)**, *IRF3* **(B)**, *IRF7* **(C)**, *Mx* **(D)**, *RSAD2* **(E)**, and *ISG56* **(F)** by qRT-PCR analysis. The results were calculated by normalization to the expression of *L. crocea β-actin* and then recorded as fold change compared to the control group. All data are expressed as the mean of three individual experiments, with bars representing the SE. *P < 0.05; **P <0.01; NS, not significant.

To further elucidate the potential effects of *Lc*-SARM on the downstream immune-related genes expression, the mRNA expression patterns of large yellow croaker antiviral-related genes such as *IFN1*, *IRF3*, *IRF7*, *Mx*, *RSAD2*, and *ISG56* under the only overexpression and also the co-expression of *Lc*-SARM with the molecules mentioned above were examined. The results showed that *Lc*-SARM overexpression could significantly up-regulate the mRNA expression levels of *IRF3*, *IRF7*, and *RSAD2*, but not *IFN1*, *Mx*, and *ISG56* in comparison with the control cells transfected with the empty vector (**Figures 12A-F**). The co-expression of *Lc*-SARM with *Lc*-TRIF significantly down-regulated the mRNA expression levels of all the six detected antiviral-related genes in contrast to the only overexpression of *Lc*-TRIF (**Figures 12A-F**). Under the co-expression of *Lc*-SARM with *Lc*-TRAF3, the transcriptional levels of *IFN1* and *IRF3* were significantly reduced compared to the overexpression of *Lc*-TRAF3 alone (**Figures 12A, B**), and the expression level of *IRF7* was also significantly down-regulated compared to the only overexpression of *Lc*-SARM (**Figure 12C**), whereas the expression levels of *Mx*, *RSAD2*, and *ISG56* were not significantly affected (**Figures 12D-F**). In addition, the expression levels of *IRF3*, *Mx*, and *RSAD2* were significantly down-regulated in *Lc*-SARM and *Lc*-IRF3 co-expressed cells compared to the overexpression of *Lc*-IRF3 alone (**Figures 12B, D, E**), with the expression level of *IRF7* also been found significantly down-regulated compared to the only overexpression of *Lc*-SARM (**Figure 12C**). However, the co-expression of *Lc*-SARM with *Lc*-IRF3 were not significantly affected the mRNA expression level of *IFN1* and *ISG56* (**Figures 12A, F**). Moreover, in comparison with the overexpression of *Lc*-IRF7 alone, the expression levels of *IFN1*, *IRF7*, *Mx*, and *ISG56* were significantly down-regulated in *Lc*-SARM and *Lc-*IRF7 co-expressed cells (**Figures 12A, C, D, F**), and compared to the overexpression of *Lc*-SARM alone, the expression patterns of *IRF3* and *RSAD2* were also significantly abolished in the cells that co-expressed *Lc*-SARM with *Lc-*IRF7 (**Figures 12B, E**).

## 4 Discussion

As a TIR domain-containing protein, SARM has been identified as an adaptor and plays a negative regulation in TLR-mediated signaling (13–15), other studies have also demonstrated the regulate function of SARM in the apoptosis of mammals (16, 17). In this study, the ortholog of SARM was characterized in large yellow croaker, the interaction of SARM with TRIF, TRAF3, IRF3, and IRF7, the association of such molecules in the activation of NF-κB, IRF3, IRF7, and type I IFN promoters, as well as in the antiviral responses and the induction of downstream immune-related genes expression were also investigated, which revealed the involvement of SARM in regulation of the host immune-related signaling in teleosts.

The expression of *Lc-SARM* was widely distributed in all the detected organs/tissues of the healthy fish, with the highest expression level found in the brain, which was to some extent consistent with the previous clues that SAMR functions in the development of olfactory neurons in the worm like *C. elegans* (12), and mouse SARM chiefly expression in the brain and could play a role in regulation of the neuronal death (39). It is thus assumed that teleost SARM may also play similar roles in the neuron development or survival, although few reports have presented till now. In addition, the previous studies have reported that the SARM ortholog in *C. elegans*, termed TIR-1, was crucial for the immune responses in treating bacterial infections (10, 11). Moreover, studies in the “living fossil” horseshoe crab (*Carcinoscorpius rotundicauda*) revealed the *SARM* ortholog could be rapidly up-regulated under *Pseudomonas aeruginosa* infection (40), another report in white shrimp (*Litopenaeus vannamei*) also showed that *SARM* could be significantly up-regulated after *Vibrio alginolyticus* as well as white spot syndrome virus (WSSV) infections (41). Compare to the reports in invertebrates, studies in teleosts indicated that grass carp *SARM* could be induced under GCRV or viral/bacterial PAMPs stimulations *in vivo* and *in vitro* (23), and the expression level of *SARM* was also up-regulated in the isolated head-kidney lymphocytes (HKLs) of mandarin fish under poly(I:C) and LPS challenge (24). Consistent with these findings, our presented results also revealed that the expression level of *Lc-SARM* could be significantly induced in response to the stimulations of the viral/bacterial PAMPs, including poly I:C, LPS, and PGN, and also the infection of *P. plecoglossicida*. It is thus speculated that SARM ortholog plays important roles in the host immune responses in vertebrates as well as that in invertebrates, although the exact function may be varied from different species.

In mammals, SARM was determined as a negative regulator in MyD88- and TRIF-dependent pathways, which was dependent on the direct TIR-TIR interaction with MyD88 and TRIF in mammals (13, 14). In teleost, mandarin fish SARM could interact with MyD88 and TRIF to impair the host antiviral signaling (24), and grass carp SARM could down-regulate the mRNA expression level of immune-related genes during the TRIF- and MyD88-mediated signaling pathways, thus suppressing type I IFN production in response to GCRV infection (23). Similarly, our present results also showed that *Lc*-SARM could interact with *Lc*-TRIF. Although *Lc*-SARM overexpression could significantly induce the activation of NF-κB, IRF3, IRF7, and type I IFN promoters, the co-expression of *Lc*-SARM with *Lc*-TRIF significantly impaired *Lc*-TRIF mediated NF-κB, IRF3, IRF7, and type I IFN promoter activation. In addition, in contrast to the overexpression of *Lc*-TRIF alone, *Lc*-SARM co-expressed with *Lc*-TRIF could significantly abolish the antiviral response against SVCV infection, and down-regulated the mRNA expression level of antiviral-related genes, including *IFN1, IRF3, IRF7, Mx, RSAD2*, and *ISG56*. It is thus collectively indicated that SARM acts as an important negative regulator in TRIF-mediated signaling in teleosts, which is consistent from that in mammals.

TRAF3, as an important member of TRAF family, has been demonstrated to play important roles in type I IFNs production in TRIF-mediated signaling (42). In our previous investigations in large yellow croaker, *Lc*-TRAF3 could significantly up-regulate *Lc*-TRIF-mediated NF-κB and IRF3 promoter activation (29). Intriguingly, our present results showed that *L*c-SARM could form a protein complex with *Lc*-TRAF3, and co-expression of *L*c-SARM with *Lc*-TRAF3 significantly reduced the luciferase activity of NF-κB and IRF3 promoter in comparison with the only overexpression of *Lc*-TRAF3, and the IRF7 promoter activity was also significantly impaired in *L*c-SARM and *Lc*-TRAF3 co-expressed cells compared to the only overexpression of *L*c-SARM. Meanwhile, the antiviral response of *Lc*-TRAF3 mediated against SVCV infection in EPC cells was impaired when *Lc*-SARM co-expressed with *Lc*-TRAF3, and the transcriptional levels of *IFN1* and *IRF3* were also significantly down-regulated in *Lc*-SARM and *Lc*-TRAF3 co-expressed LYCMS cells compared with the overexpression of *Lc*-TRAF3 alone. It is thus suggested that *L*c-SARM could also abolish *Lc*-TRAF3-mediated signaling, which is a novel finding in teleosts.

IRF3 and IRF7, as the pivotal transcription factors, function essentially in TRIF-mediated signaling and play important roles in triggering the production of type I IFNs (43). Our present results showed that *Lc*-SARM could interact with *Lc*-IRF3 and *Lc*-IRF7. Notably, *Lc*-SARM co-expressed with *Lc*-IRF3 could significantly abolish the NF-κB, IRF3, IRF7, and type I IFN promoter activation compared to the only overexpression of *Lc*-SARM or *Lc*-IRF3, and the co-expression of *Lc*-SARM with *Lc*-IRF7 could also significantly impair the NF-κB, IRF3, and IRF7 promoter activation. Furthermore, the co-expression of *Lc*-SARM with *Lc*-IRF3 and *Lc*-IRF7 could significantly suppress *Lc*-IRF3 and *Lc*-IRF7 involved antiviral responses against SVCV infection in EPC cells, the co-expression of *Lc*-SARM with *Lc*-IRF3 could also significantly impair the expression levels of *IRF3*, *Mx*, and *RSAD2* in contrast to the overexpression of *Lc*-IRF3 alone, and the co-expression of *Lc*-SARM with *Lc*-IRF7 could clearly reduce the expression of *IFN1*, *IRF7*, *Mx*, and *ISG56* in comparison with the overexpression of *Lc*-IRF7 alone. These findings collectively suggested that *Lc*-SARM functions as a negative regulator in *Lc*-IRF3/7-mediated signaling. To our knowledge, it is the first report that vertebrate SARM could associate with IRF3 and IRF7 in regulation of the host immune responses, and the inhibition of such signaling is through a direct interaction with IRF3 and IRF7.

**Figure 13:**
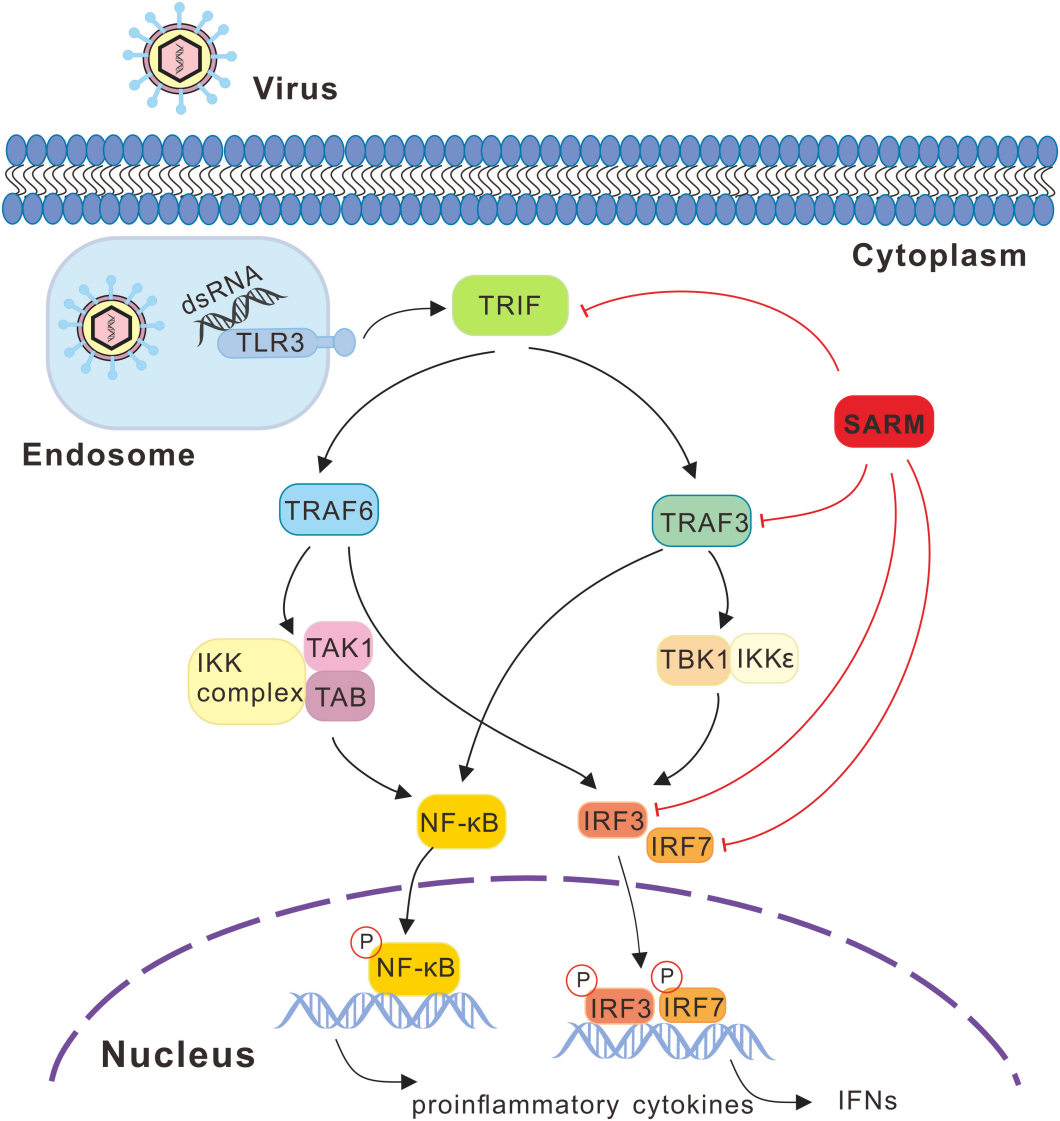
SARM mediated signaling pathway in response to viral infection in large yellow croaker. Recognition of viral PAMPs such as dsRNA through TLR3, which then recruits the adaptor protein TRIF, subsequently actives the downstream TRAF3 and TRAF6, leading to the activation of transcription factors including NF-κB and IRF3/7 and then initiating the production of proinflammatory cytokines and type I IFNs. During the signaling cascade, large yellow croaker SARM could associate with TRIF, TRAF3, IRF3, and IRF7 and acts as an inhibitor in such molecules mediated signaling. In the signaling schematics, the involved signaling pathway is presented with lines and arrows, with the negative regulation cascades marked with red lines.

In summary, the present study characterized a *SARM* ortholog in large yellow croaker, which could be significantly induced under the poly I:C, LPS, PGN, and *P. plecoglossicida* stimulations. In particular, although *Lc*-SARM could induce NF-κB, IRF3, IRF7, and type I IFN promoter activation, the co-expression of *Lc-*SARM with *Lc-*TRIF, *Lc*-TRAF3, *Lc*-IRF3, or *Lc*-IRF7 significantly abolished the induction of NF-κB, IRF3, IRF7, or type I IFN promoter activation, and also suppressed *Lc-*TRIF, *Lc*-TRAF3, *Lc*-IRF3, and *Lc*-IRF7 mediated antiviral response by inhibiting some of the antiviral-related genes expression. Such regulation was through a physical interaction between *Lc*-SARM and *Lc-*TRIF, *Lc*-TRAF3, *Lc*-IRF3, and *Lc*-IRF7. It is thus suggested that *Lc*-SARM functions as a negative regulator in *Lc-*TRIF, *Lc*-TRAF3, *Lc*-IRF3, and *Lc*-IRF7 involved signaling (**Figure 13**), further studies should clarify the exact mechanism that involved.

## Data availability statement

The original contributions presented in the study are included in the article/**Supplementary Material**. Further inquiries can be directed to the corresponding authors.

## Ethics statement

The animal study was reviewed and approved by Animal Administration and Ethics Committee of Jimei University (Permit No. 2021-4).

## Author contributions

PZ conceived and designed the research. PZ, JZ, YL, JT, KL, JS, and CL performed the experiments and analyzed the data. PZ, JZ, and YL wrote the manuscript. PZ, YL, YJ, ZZ, and YW revised the manuscript. All authors contributed to the article and approved the submitted version.

## Supplementary material

Supplementary Figure 1 Multiple alignment of the amino acid sequence of *Lc-*SARM with those of other vertebrate SARM. Comparison of *Lc*-SARM with other vertebrate SARM by using Clusta X and GeneDoc program. All the sequences share the same amino acid residue are shown in the black shaded areas, and the conservative and semi-conservative amino acid substitutions are represented in the gray and light gray shaded areas, respectively. The ARM domain, two SAM domains, and the TIR domain are indicated with black arrows.
