# Supplementary figures and images for: SARM suppresses TRIF, TRAF3, and IRF3/7 mediated antiviral signaling in large yellow croaker Larimichthys crocea
Source: Front Immunol. 2023 Jan 13;13:1021443. doi: 10.3389/fimmu.2022.1021443 (PMC9880191; doi:10.3389/fimmu.2022.1021443)

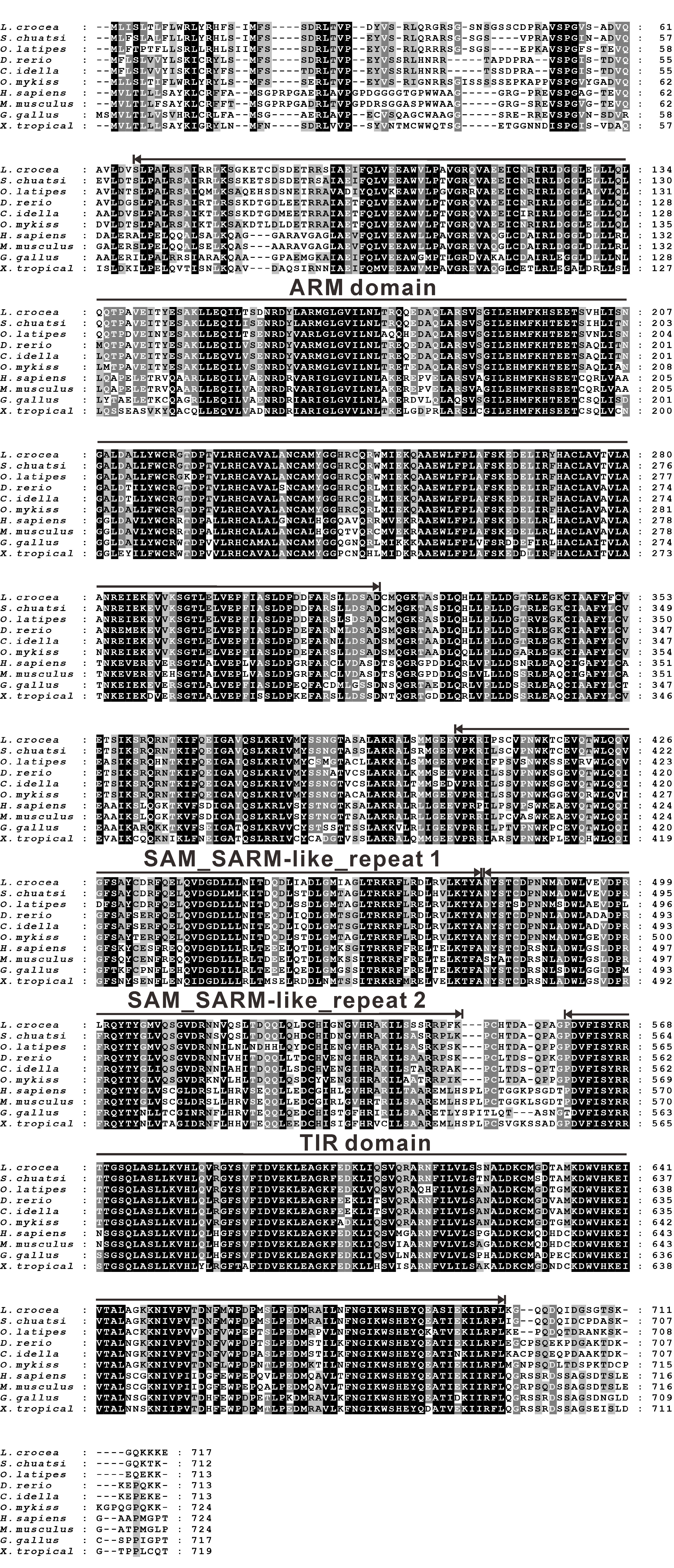

Supplement: Supplementary Figure 1 — Multiple alignment of the amino acid sequence of Lc-SARM with those of other vertebrate SARM. Comparison of Lc-SARM with other vertebrate SARM by using Clusta X and GeneDoc program. All the sequences share the same amino acid residue are shown in the black shaded areas, and the conservative and semi-conservative amino acid substitutions are represented in the gray and light gray shaded areas, respectively. The ARM domain, two SAM domains, and the TIR domain are indicated with black arrows. [file Image_1.tif]
